# Dietary Guidance, Sensory, Health and Safety Considerations When Choosing Low and No-Calorie Sweeteners

**DOI:** 10.3390/nu17050793

**Published:** 2025-02-25

**Authors:** John L. Sievenpiper, Sidd Purkayastha, V. Lee Grotz, Margaux Mora, Jing Zhou, Katherine Hennings, Cynthia M. Goody, Kristen Germana

**Affiliations:** 1 Department of Nutritional Sciences, Temerty Faculty of Medicine, University of Toronto, Toronto, ON M5S 1A1, Canada; john.sievenpiper@utoronto.ca; 2 Department of Medicine, Temerty Faculty of Medicine, University of Toronto, Toronto, ON M5S 1A1, Canada; 3 Division of Endocrinology and Metabolism, Department of Medicine, St. Michael’s Hospital, Toronto, ON M5B 1W8, Canada; 4 Toronto 3D Knowledge Synthesis and Clinical Trials Unit, Clinical Nutrition and Risk Factor Modification Centre, St. Michael’s Hospital, Toronto, ON M5B 1W8, Canada; 5 Li Ka Shing Knowledge Institute, St. Michael’s Hospital, Toronto, ON M5B 1W8, Canada; 6 SP Advisors Inc., Chicago, IL 60605, USA; siddp@sp-advisor.com; 7 Department of Food Science, University of Massachusetts, Amherst, MA 01003, USA; 8 ToxInsight, LLC, Fort Washington, PA 19034, USA; vlgtoxinsight@gmail.com; 9 Ingredion Inc., Bridgewater, NJ 08807, USA; margaux.mora@ingredion.com (M.M.); kristen.germana@ingredion.com (K.G.); 10 PureCircle by Ingredion Inc., Westchester, IL 60154, USA; katherine.hennings@ingredion.com; 11 Grow A Food Career, LLC, Elmhurst, IL 60126, USA; growafoodcareer@gmail.com

**Keywords:** low and no-calorie sweeteners, dietary guidance, sensory, health, safety

## Abstract

The growing global focus on the adverse health conditions associated with excessive sugar consumption has prompted health and policy organizations as well as the public to take a more mindful approach to health and wellness. In response, food and beverage companies have proactively innovated and reformulated their product portfolios to incorporate low and no-calorie sweeteners (LNCSs) as viable alternatives to sugar. LNCSs offer an effective and safe approach to delivering sweetness to foods and beverages and reducing calories and sugar intake while contributing to the enjoyment of eating. The objective of this paper is to enhance the understanding of LNCSs segmentation and definitions, dietary consumption and reduction guidance, front-of-package labeling, taste and sensory perception and physiology, metabolic efficacy and impact, as well as the overall safety of LNCSs and sugar.

## 1. Introduction

Globally, public health organizations, policy and regulatory agencies, and consumers have become more focused on poor health outcomes associated with excess sugar consumption. With the public’s actions towards a more mindful health and wellness lifestyle, food and beverage companies continue to evolve their product portfolios with low and no calorie sweeteners (LNCSs) as a sugar replacement.

As an effective and safe approach to deliver sweetness to foods and beverages, LNCSs aids in decreasing caloric and sugar intake while contributing to eating enjoyment. Known as having greater sweetness compared to sucrose (table sugar), lesser amounts of LNCSs in foods succeed in delivering a similar level of sweetness, leading to an individual’s reduction in calories and sugar.

The purpose of this paper, relevant to LNCSs and sugar, is to augment the understanding of segmentation and definitions; dietary consumption and reduction guidance; front-of-food package labeling; taste and sensory perception and physiology; metabolic efficacy and impact as well as overall safety of use.

## 2. Sweeteners Segmentation and Definitions [1,2,3,4]

**Figure 1:**
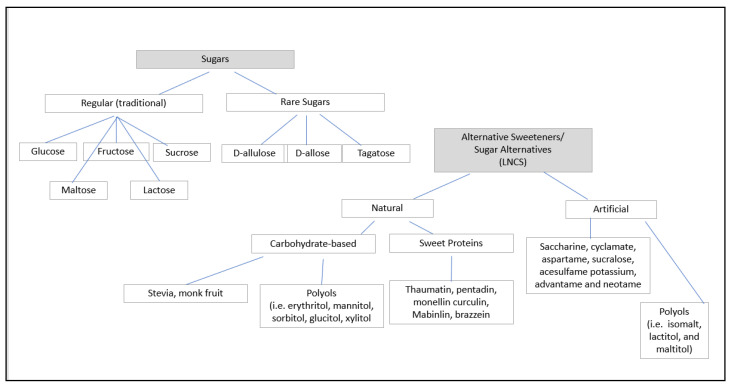
Schematic for Categorizing Sweeteners.

Sweetening ingredients can be classified as sugar, which include regular sugars and rare sugars, and alternative sweeteners. Figure 1 depicts and suggests a schematic for how to categorize sweeteners relative to sugar and LNCSs. Alternative sweeteners are typically further segmented into “natural” and “artificial,” however these terms are not clearly defined by regulatory agencies. Malaysia MOH (2004) has included stevia extract as a subgroup of sugar and other caloric sweeteners derived from plants. Thus, stevia extracts (Reg 118A) are claimed as natural sweeteners along with sugar, brown sugar, and dextrose; other synthetic sweeteners are classified under the artificial sweeteners group [5]. The Health Ministry of Indonesia divided the Pemanis (sweetener) group into Pemanis Alami (Natural sweetener) and Pemanis Butani (artificial sweetener). Pemanis Alami includes steviol glycosides [6]. The Korean Food Additive included stevia extract under “Natural Food Additive” [7]. For the purposes of this review, sweeteners that can be found in nature are referred to as natural and those that are not found in nature are considered artificial.

### 2.1. Sugars

Sugars are monosaccharide and disaccharide carbohydrates that are soluble in water and provide sweetness, bulk and calories. Depending on the abundance in the natural source, sugars can be classified as regular (traditional) and rare sugars.

### 2.2. Regular Sugars

The main regular sugars are glucose, fructose, sucrose, maltose, and lactose, which provide sweetness and contribute approximately 4 kcal/g. The total sugars in food and beverages are made of added sugars, intrinsic sugars, and milk sugars. Added sugars are used by manufacturers and consumers to sweeten food and beverages. Intrinsic sugars are naturally contained within fruits and vegetables. Milk sugars are in milk. Other commercially available forms of sugars include honey, maple syrup, rice syrup, high fructose corn syrup (HFCS) and caramel syrup.

### 2.3. Rare Sugars

Found in small quantities in nature, rare sugars have slight differences in their chemical structure compared with regular sugars. The commercially viable rare sugars are D-allulose and D-tagatose, which provide approximately 70% and 92% sweetness of sugars with 0.4 and 1.5 kcal/g, respectively. The caloric values for labeling vary by country. Allulose is the epimers of fructose. Tagatose is an isomer of fructose. Other examples of rare sugars include allose, arabinose and xylose. The rare sugars provide bulk to reduced-sugar food and beverages prepared with low or no-calorie sweeteners.

### 2.4. Alternative Sweeteners

Alternative sweeteners are sugar replacers and consist of non-caloric (non-nutritive) and caloric sweeteners that are found in nature or must be synthesized in a lab. The LNCSs (low-no calorie sweeteners) include non-nutritive sweeteners (NNSs) and low-calorie sweeteners (LCSs). The non-nutritive sweeteners provide a very high degree of sweetness compared to sucrose and contribute no calories to sweeten a food product. Low-calorie sweeteners generally contribute lower calories and lower sweetness compared to sucrose. Additionally, NNSs provide no bulk in food applications since their usage level is very small. LCSs are used for providing bulk in food application with a modest level of sweetness.

LNCSs, found in nature, can be subdivided into three groups: 1.sweet proteins such as thaumatin (katemfe fruit), pentadin (oubli plant), monellin (serendipity berry), curculin (fruit of Curculigo latifolia), Mabinlin (seed of mabinlang), and brazzein (oubli climbing plant); 2. carbohydrate-based sweeteners including stevia and luo han guo (monk fruit); and 3. polyols or sugar alcohols, which are a group of natural sweeteners with fewer calories and a lower sweetness than sugars that add bulk to food. Erythritol, mannitol, sorbitol, glucitol and xylitol are found naturally in fruits and vegetables Manmade polyols include isomalt, lactitol, and maltitol.

Sugar alcohols are defined as the saccharide derivatives in which a hydroxyl group replaces a ketone or aldehyde group. Major commercial sugar alcohols are erythritol, sorbitol, maltitol, mannitol, xylitol, lactitol, and isomalt. Sugar alcohols have defined intake limits to mitigate effects related to over consumption. They are only partially absorbed in the gut and due to their osmotic effects, polyols draw fluid into the large intestine. When consumed in excess amounts, polyols may cause a laxative effect. In fact, mannitol is commercially sold over the counter for health and wellness purposes.

Artificial sweeteners are manufactured sweeteners and are not generally found in nature. They are also commonly referred to as ‘high potency’ sweeteners and include saccharin, cyclamate, aspartame, sucralose, acesulfame potassium, advantame and neotame. Artificial sweeteners provide no bulk to food and drinks.

Key Points

- Sweetening ingredients in food and beverages can be divided into sugars and alternative sweeteners.
- Regular sugars contribute approximately 4 calories/g and consist of monosaccharide and disaccharide sugars from natural sources.
- Rare sugars are available in limited quantity from natural sources, and they provide lower sweetness with less than 4 calories/g. Both regular and rare sugars contribute bulk to food and beverages.
- Alternative sweeteners include low- and no- calorie sweeteners. They are either natural or artificial sweeteners with little or no caloric contribution. The no-calorie sweeteners are higher in sweetness potency than sugar but provide no bulk to food and drinks. The low-calorie sweeteners are polyols or sugar-alcohols that contain fewer calories and a lower sweetness than sugars, but they provide bulk to foods and beverages.
- Total sugars in food and beverages are made of added sugars, intrinsic sugars, and milk sugars. Added sugars are used by manufacturers and consumers to sweeten food and beverages. Intrinsic sugars are naturally contained within fruits and vegetables. Milk sugars are in milk.

## 3. Dietary Guidance for Sugar Consumption and Reduction

Improving diets to reduce obesity, diabetes, chronic illnesses, and dental caries is a global priority in the context of sugar intake. Globally, the association between dietary sugar consumption and its approaches for reduction continue to affect public health outcomes [8,9,10]. Food-based dietary guidelines issued by leading global authorities offer sugar consumption guidance classified as either total sugars, added sugars and free sugars [10,11,12,13,14]. As depicted in Table 1, these terms are either quantified as a daily intake amount of sugar or qualified as consuming the least amount of sugar per day.

Complementing the sugar consumption, guidance offered by the WHO and others, of the most populated countries in each of the six global regions (Asia, Australia/Oceania, Europe, North America, South America, and Africa), 25 in total presented in Table 2. The majority have qualitative and/or quantitative recommendations for sugar consumption among adult, child, and pregnant populations [8,9,10,11,12]. Certain countries forego offering recommendations. Examples of the recommendations in action range from a definitive limit on consumption of 25 g of free sugars per day to several five-gram portions of sugar based on physical activity and encouraging the public to drink water to sparingly consume food and drinks with added sugars [8,9,13,16,17,18,19,20,21,22,23,24,25].

In addition to global health agencies providing sugar consumption guidance, organizations committed to supporting individuals with diabetes offer positions about LNCSs in the diet. For individuals with diabetes and families supporting them with their eating, knowing what LNCSs may support a reduction in the consumption of sugar as well as decrease dietary caloric and carbohydrate intake as a part of the eating plan is important. As shown in Table 3, organizations dedicated to improving the well-being of people with diabetes and their families offer guidance about positioning LNCSs as a part of the diet.

### 3.1. Global Sugar Reduction Guidance Approaches

Sugar intake originates from sweets, beverages, fruits, vegetables, and dairy. Attention has focused on curtailing the sugar intake of these products. Solutions range from government guidelines, consumer behavior changes, industry formulation, marketing restrictions, and taxation [14,29,30,31,32,33,35,36,37,38,43]. 

Of the possibilities for sugar reduction, the Global Nutrition Report monitors and tracks the presence of a country-level sugar-sweetened beverage tax. More than half of the 25 countries examined in this review have enacted a sugar-sweetened beverage (SSB tax) [9].

### 3.2. Agency and Government Guidelines

Globally, mandatory and voluntary guidelines have informed policy and program development and industry formulation in assessment, guidance, planning, implementation, and evaluation of the current intake levels of sugar. Examples of these activities include consumption guidelines noted earlier as well as promoting increased water consumption, campaigning for fruit and vegetable campaigns, and delivering consumer education about sugar labeling and sweetener substitutes in a variety of food and beverage products [44,45,46,47,48,49,50,51,52,53,54,55,56,57,58].

### 3.3. Consumer Behavior Changes

Consumers in some areas of the world are rethinking their food behaviors and choices as they relate to consumption of sugars and the impact on their health. This, in combination with the abundance of information available, individuals are thoughtfully considering their sugar intake and reduction strategies. Conversely, consumers in vulnerable global regions may be unable to change behavior due to geo-political public health infrastructure. Further, measuring attitudes and cognition influencing the effectiveness of representative population-based and individual behaviors presents geographic and resource challenges [59,60,61].

One approach to facilitate consumer behavior with food choices is the application of front-of-package (FOP) food labeling. The idea is to enable accessible and transparent information when making choices at the point of sale. What remains unknown is the effectiveness and outcomes associated with nutrition education on packaging. Additional details about FOP are presented later in this paper.

Another behavior change is to limit or avoid sugar. According to the 2023 International Food Information Council (United States) Food and Nutrition Survey, 72% of respondents reported either limiting or avoiding sugar entirely. Among those surveyed, sugar remained preferred over low calorie and no calorie sweeteners. However, one of the common benefits cited for using low calorie and no calorie sweeteners included reducing sugar intake without added calories [52].

Further, consumers modify behaviors by decreasing their sugar intake as a part of daily activities. Based on sugar reduction strategy documents from Australia, New Zealand, the United Kingdom and the United States, a total of 1145 strategies were identified from 47 internet sources [53]. Content from the reduction strategy documents focused on informing decisions to decrease sugar intake. With the abundance of guidance from a variety of sources, consumers in various geographic areas seek support to change their sugar intake.

### 3.4. Industry Formulation

Common approaches by the food industry to reduce sugar in food and beverages include innovating smaller packaging sizes, promoting products with less sugars, and reformulating with less sugar and/or replacing with low to no calorie sweeteners in the original product size. However, demonstrating progress requires time. To acclimate and accommodate consumers’ taste for less sweetness over time, consumer-packaged goods companies proceed slowly in reducing sugars. If not, they risk facing consumers’ rejection of the product. The path of reducing sugars while maintaining sweetness remains a challenge [56,57].

### 3.5. Global and Country-Specific Marketing Restrictions of Sugar

To address childhood obesity and its impact on adulthood, the WHO, as well as other global and local regulatory and public policy makers, has regulated advertising and marketing practices of high fat, salt and sugary (HFSS) foods and beverages to children. In 2023, the WHO released new guidelines recommending countries implement comprehensive mandatory policies to protect children of all ages from the marketing of foods and non-alcoholic beverages that are HFSS [59,60,61,62,63].

Examples of existing policy-based country-level marketing restrictions include the following:

- Mexico—Restricts television advertising of certain foods to audiences of more than 35% children during certain weekday and weekend hours [64].
- The United Kingdom—Bans sugary food and drink advertising during children’s TV programs. Rules exist when engaging celebrities and licensed characters appealing to kids in unhealthy food marketing [59,60,61].
- The United States—As a part of the Better Business Bureau, the Children’s Food and Beverage Advertising Initiative (CFBAI) food industry participants voluntarily commit that in advertising primarily directed to children, they will either not advertise foods or beverages to children at all or advertise only products that meet CFBAI’s strict Uniform Nutrition Criteria. Participants also do not advertise in elementary schools [65].

### 3.6. Taxation

Sugar-sweetened beverages (SSBs) appear to be one of the most taxed product categories globally. As of July 2022, at least 108 countries worldwide apply national-level taxes to at least one type of SSB [54]. The efficacy of and motivations for taxation vary. As shown in Table 2, more than half of the 25 countries examined in this review have enacted a sugar-sweetened beverage (SSB tax). Evidence suggests that SSB taxes are an effective intervention to increase and promote reductions in the intake of sugar as well as improve the public’s health and forego costs of healthcare [55,56]. 

An example of establishing a national health policy to address the excessive overweight and obesity prevalence in Mexico is the tax of one Mexican peso per liter of sugar-sweetened beverage (SSB) that came into effect in 2014. SSB purchases decreased, and water purchases increased after an SSB tax was imposed in Mexico [66].

To conclude, not all countries conducted sugar consumption surveys in recent years. Walton and colleagues presented a picture of global sugar consumption levels [67]. Based on data with adults, for the countries with data available for free sugar consumption, most did not meet the WHO’s guideline of <10% total energy intake and none met the <5% total energy recommendation for additional health benefits. In addition, developed countries like the United States and the United Kingdom’s added sugar and free sugar consumptions, respectively, exceeded their national dietary guidelines. It is apparent that gaps exist between sugar consumption and dietary guidelines globally, and this is an opportunity for food and beverage manufacturers to formulate innovatively to support dietary guidelines and population health.

Without question and in the context of the public’s health and related outcomes, sugar consumption and its reduction in foods and beverage remain top of mind with government regulators, food manufacturers, health and medical professionals and academicians. Efforts have been dedicated to assessing and evaluating sugar intake of adults and children followed by policy development in the arena of dietary-based food and beverage consumption of sugar. Mandatory and voluntary programs exist to facilitate action by the food industry and create educational awareness for a change in sugar consumption across communities and populations. Reduction guidance for dietary sugars emphasizes choosing products with less sugars; limiting specific food and/or beverage consumption; and preparing foods with less sugars. Nations without sugar consumption guidance and approaches to reduction may not have the geo-political infrastructure and data to develop policies and programs and may rely on globally recognized guidance and/or forego offering recommendations.

In the context of current public health outcomes associated with sugar consumption, a critical need exists to develop a standardized system for identifying and quantifying added sugars across the food supply chain. Unified communications from trusted sources are essential to increase consumer awareness and drive positive behavioral changes related to reducing sugar intake.

Key Points

- Global and national sugar consumption guidelines inform the development of public health policy and programs focused on the reduction in dietary sugar.
- Improving diets to reduce obesity, diabetes, chronic illnesses, and dental caries is a global priority in the context of sugar intake.

## 4. Front-of-Package Labeling Landscape: Global and Local

Front-of-package (FOP) food labeling plays a crucial role in informing consumers about the nutritional content and health attributes of packaged food products. As consumers become increasingly health-conscious and seek transparency in food choices, the global landscape of FOP labeling has witnessed significant developments and variations. Here, we have identified some key trends, challenges, and regulatory approaches shaping the FOP labeling landscape worldwide. Table 4 provides a summary of current FOP labeling schemes by country.

### 4.1. Key Trends

#### 4.1.1. Nutrient-Specific Labeling

**Figure 2:**
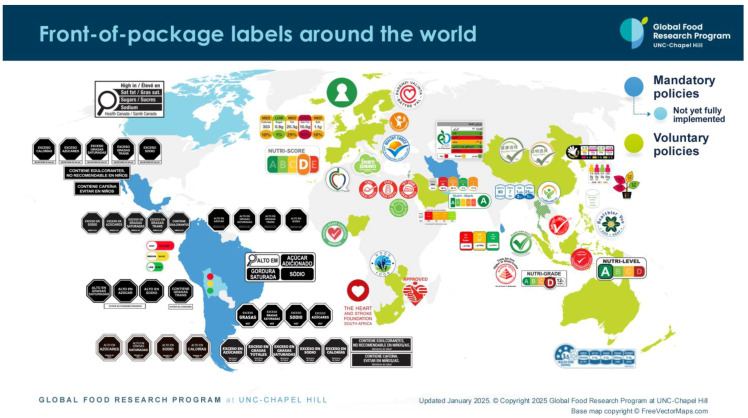
Front-of-package labels around the world. Reprinted with permission from the Global Research Program at UNC-Chapel Hill.

As shown in Table 4 and Figure 2, many countries are adopting nutrient-specific labeling which prominently displays key nutritional information such as calories, saturated fats, sugars, and sodium on the front of food packages. Many times, this FOP labeling scheme involves the use of nutritional warnings that use text-based seals to inform consumers when a product contains excess amounts of critical nutrients. This nutrient-specific approach aims to provide consumers with quick and easily comprehensible information when making choices on what to purchase or consume.

#### 4.1.2. Traffic Light System

The traffic light system, using color-coded labels to indicate the levels of key nutrients, has gained popularity in various regions. With this scheme, red is used to indicate a high level of an undesirable nutrient content, yellow for moderate, and green for low.

#### 4.1.3. Health Claims and Symbols

Some FOP labels include health claims and symbols endorsed by health organizations or regulatory bodies. These symbols convey that a product meets specific nutritional standards, helping consumers make informed decisions about the healthfulness of a particular item.

### 4.2. Challenges

#### 4.2.1. Global Harmonization

Lack of global harmonization in FOP labeling poses challenges for both consumers and manufacturers. Varying standards and formats make it difficult for consumers to compare products across different regions, and manufacturers face the burden of complying with multiple labeling requirements.

#### 4.2.2. Consumer Understanding

Ensuring that consumers understand and interpret FOP labels accurately is a persistent challenge. The diversity of labeling systems, symbols, and terminology can lead to confusion, potentially hindering the effectiveness of FOP labeling in promoting healthier choices.

### 4.3. Regulatory Approaches

#### 4.3.1. Government Regulations

Many countries have implemented or are considering government regulations to standardize FOP labeling. These regulations define the format, content, and criteria for labeling, aiming to create a consistent system that facilitates consumer understanding.

#### 4.3.2. Industry Initiatives

In response to the demand for clearer labeling, some food manufacturers and retailers have voluntarily adopted front-of-pack labeling schemes. As depicted in Table 4, these initiatives often involve the use of interpretive labels, symbols, or logos that provide a quick visual reference for consumers.

Studies to evaluate the effectiveness of front-of-pack labeling in informing consumers about the nutritional content of food products and influencing their purchasing behavior vary in methodology, scope and the specific type of FOP labeling evaluated. Key findings suggest FOP rating systems or symbols may help consumers identify healthy foods and consumers are more likely to notice and comprehend FOP labels compared to traditional nutrition labels on the back of packages. Studies also indicate consumers prefer FOP labels that are simple, easy to understand and consistent across various products and brands. It is also clear that FOP labeling has led manufacturers to reformulate products for a more favorable nutrient profile.

What remains unclear in the scientific literature is the effectiveness of FOP labels in changing consumers’ purchase intention. Further, the information on FOP labels contributes to healthier food purchases remains inconclusive. Additional research is needed to understand whether the use of these labels results in a consumption of healthier diets and better overall health outcomes.

Key Points:

- The global landscape of FOP food labeling reflects a dynamic interplay between industry initiatives and government regulations.
- While trends like nutrient-specific labeling and the traffic light system are becoming widespread, challenges such as global harmonization, and ensuring consumer comprehension persist.
- As the landscape continues to evolve, collaboration between governments, industry stakeholders, and public health advocates will play a pivotal role in shaping the future of front-of-package food labeling.

## 5. Impact of Non-Nutritive Sweeteners on Sugar Reduction: Taste and Sensory Perception

FOP labeling can impact consumer perception of products before there is a chance to taste the products. Taste is crucial for the acceptance and enjoyment of food. The sensory perception of taste plays an important role for the social and physiological well-being of humans. Of the five basic tastes (sweet, umami (savory), salty, sour, and bitter) of food [103,104], sweet remains the most prominent and desired taste across all ages.

### 5.1. Taste Perception and Physiology

Taste perception occurs when a compound interacts with a taste receptor cell to initiate a signaling cascade that sends information to the brain. The path of sensation to perception begins in the oral cavity where taste buds are housed. Humans have taste buds which contain 50–100 taste receptor cells where chemical compounds are detected and signals are transmitted [105]. Sweet, umami, and bitter taste transduction mechanisms follow stimulation of a G protein coupled receptor (GPCR) which signals phospholipase Cβ2 to activate TRPM5 which causes membrane depolarization and neurotransmitter release [103,106]. The published taste transduction receptors are hT1R2/hT1R3 heterodimer for sweet, hT1R1/hT1R3 for umami, and the T2R family of 25 taste receptor genes for bitter taste. Salty and sour tastes are sensed by channels rather than GPCRs with the epithelial sodium channel (ENaC) sensing salty taste, and Otop1 senses both strong and weak acids [103].

**Figure 3:**
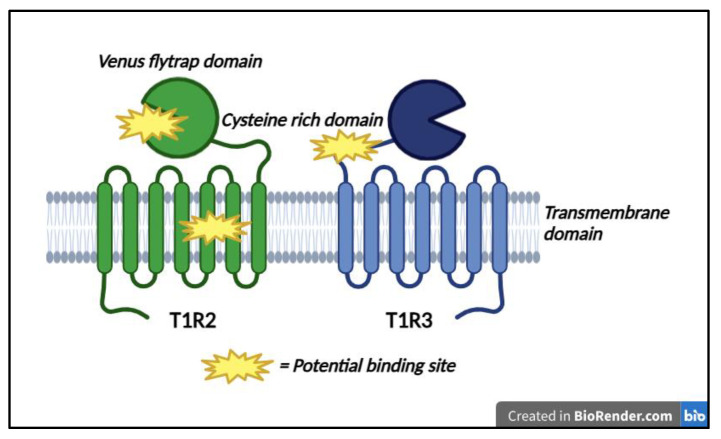
Sweet taste receptor protein and potential binding sites for sweet tasting molecules. Molecules can bind to the Venus flytrap, cysteine-rich, or transmembrane domains of the T1R2 or T1R3 to initiate sweet taste signaling. Figure created using biorender.com.

The sweet taste receptors (T1R2/T1R3) can be further divided into distinct structural domains: a Venus flytrap domain (VFTD), a cysteine-rich domain (CRD), and a seven-helix transmembrane domain (TMD) (Figure 3). Each of these domains provides potential binding site(s) for sweet compounds to interact and activate signaling cascades. Binding to the sweet taste receptor, sucrose begins a signaling cascade that stimulates an increase in Ca^2+^ to depolarize cells, release ATP, and communicate with afferent gustatory nerves [107]. Similarly, LNCSs and other caloric carbohydrate sweeteners are capable of stimulating the sweet taste receptor. However, different classes of sweet compounds bind to distinct areas of the sweet receptor (Table 5) and bind with varying affinity, which likely explains observed differences in sensory qualities between sweeteners.

### 5.2. Sensory Properties of Alternative Sweeteners

The taste of sucrose, which all other sweeteners benchmark against, is characterized by a rapid onset to peak sweetness followed by a quick decay with no apparent off flavors or bitterness. Furthermore, as concentration of sucrose increases, sweet taste intensity increases in a linear fashion [126,127]. Mechanistically, sucrose can elicit a response through two perceptual pathways, through well-defined interactions with the sweet taste receptor as well as via sodium glucose co-transporters (SGLTs) [128].

Steviol glycosides from the stevia plant have been shown to interact with bitter taste receptors hT2R4 and hT2R14 which helps to explain the bitterness and off flavors observed with glycosides at high use levels, such as Reb A, Reb C, and stevioside [119]. Apparently, Reb D and Reb M interact with these bitter receptors to a much lesser extent, resulting in superior sensory qualities. While sucralose, steviol glycosides, and monk fruit all interact with the Venus flytrap domain (VFTD) of both the T1R2 and T1R3 like sucrose, they all bind with different affinities [119]. Studies have found that the binding free energy between the hT1R2-hT1R3 and sweeteners of different compound classes shows a strong correlation with sweetness intensity for both small and large molecules [119]. Understanding these differences in binding sites gives product developers more powerful tools for improving the healthfulness of products without compromising on critical sensory characteristics.

### 5.3. Impact of Alternative Sweeteners on Satiety and Satisfaction

Upon ingestion, sucrose initiates a signaling cascade that telegraphs GI system of incoming nutrients. This cascade includes activation of the sweet taste receptor. Since LNCSs also stimulate the sweet taste receptor as sucrose, concerns have sometimes been raised on the potential impact to downstream metabolic processes. These concerns typically center around satisfaction and reward circuits as well as human compensatory behaviors when consuming LNCSs as compared to sucrose but have not been substantiated [129,130].

As discussed later in this review, LNCSs are chemically diverse compounds that, are typically unlike sugar in the way they are handled by the body. Many are largely or entirely not metabolized and are excreted unchanged following ingestion. While LNCSs are known to stimulate sweet taste receptors, they may have differential effects on satiety signaling due to the decoupling of sweet taste and calories. There can also be differences in possible effects on substances involved in satiety and appetite regulation, such as glucagon-like peptide-1 (GLP-1), peptide tyrosine (PYY), cholecystokinin (CCK), and ghrelin. For example, both caloric and noncaloric sweet compounds are sensed directly by the gut via the sweet taste GPCR and can lead to the release of GLP-1 by enteroendocrine cells, but the magnitude of the release can vary [131]. The effects seen in in vitro studies, also are not necessarily indicative of what will occur with actual consumption, as noted in a systematic review of studies with human consumption of non-nutritive sweetened beverages [132].

In addition to sweet taste receptors in the gut, bitter taste receptors are also present here and play a key role in satiety regulation. When stimulated, bitter taste receptors stimulate the release of CCK [133], however the role that NNSs play in activating these receptors and subsequent satiety signaling has not been well explored. Recent work by Noya-Leal and colleagues has demonstrated that Reb A from the stevia leaf is capable of stimulating GLP-1 release via stimulation of this bitter taste signaling pathway [134]. Again, however, interpretation of such exploratory studies demands rigor when trying to understand their impact on appetite and health. As reviewed by O’Connor et al. 2021 [135] and Adrade et al. 2021 [136], there is insufficient data to determine the degree to which LNCSs can exert an effect on the gut microbiota, adipogenesis, glycemia, appetite, or body weight in the short- or long-term. Existing works attempting to characterize the impact of LNCSs on appetite vary greatly in doses of sweeteners used, differences in study design, and use of model organisms versus humans, complicating the ability to draw conclusions on how specific sweeteners may impact appetite. RCTs assessing the impact of the chemically diverse LNCS compounds on GLP-1, GIP, CCK, and additional appetite regulating hormones is still needed to determine the role sweeteners may play in impacting appetite and satiety. Furthermore, it is unclear to what extent appetite alterations will impact sweet food cravings and subsequent potential overconsumption. Care should be taken not to assume all LNCSs will act the same, as they vary in their chemical composition. Moreover, results from in vitro studies may not translate into meaningful effects in a whole-body system, with the complex nature of satiety and appetite regulation. Additional work is needed on understanding how these very distinct molecular classes of sweet compounds can each impact satiety signaling peptides, and subsequently appetite.

Key Points:

- Taste perception of sweeteners is impacted by solubility, binding site, and affinity to the taste receptors, and interactions with components of saliva. Sweeteners can interact with both the sweet taste receptor as well as specific bitter taste receptors which lead to differences in their overall taste perception.
- The sensory properties of natural sweeteners differ from sucrose. NNSs such as stevia, monk fruit, and sweet proteins typically deliver differing temporal properties with certain non-sweet attributes as compared to sucrose. However, this can be improved by blending with other sweeteners. In the case of stevia, high purity next generation steviol glycosides (such as reb M) can deliver a cleaner sweetness with fewer non-sweet attributes but still differ from sucrose.
- NNSs can activate sweet or bitter taste receptors like sugar; however, clinical trials overall indicate no meaningful effects on overall satiety signaling. The difference in ability to trigger reward and satiety signaling across sweeteners further illustrates the need to treat NNSs as different compound classes.

## 6. Impact of Non-Nutritive Sweeteners on Obesity, Diabetes, and Cardiovascular Disease with Potential Mediation by the Gut Microbiome and Other Mechanisms

Non-nutritive sweeteners (NNSs) are used as a strategy to reduce calories from added sugars in the diet, especially those from sugar-sweetened beverages (SSBs), which are the most important source of added sugars in the diet. Their ability, as a class of additives, to deliver the intended benefit to reduce the intake of calories and sugars and contribute to downstream improvements in weight management and related cardiometabolic health has come under increased scrutiny. Systematic reviews and meta-analyses have shown mixed results. Non-nutritive sweetened beverages (NNSBs), the most important source of NNS in the diet, have shown inconsistent weight loss and improvements in cardiometabolic risk factors in randomized controlled trials (RCTs) [137,138,139]. The interpretation of RCTs, however, is highly dependent on the comparator and the calories available to be displaced by NNSBs with the pooling of caloric (e.g., SSBs) and non-caloric (e.g., water, placebo) comparators. This can lead to an underestimation the true effect of NNSBs [140,141,142], and, in turn, associations of higher risk of obesity, diabetes, and CVD in prospective cohort studies [137,138,139]. Equally, observational studies, which have been included in numerous meta-analyses reported in the literature, are at high risk of reverse causality (i.e., the consumption of an LNCS being a risk mitigation strategy in persons who are overweight, vs. consumption of an LNCS causing overweight). Residual confounding from behavioral clustering and measured and unmeasured confounding, can also lead to biased estimates [140,141,143]. Prevalent or baseline exposure assessments of NNSBs in observational studies appear especially vulnerable to these limitations [143,144]. There have been numerous calls for better research and reporting standards and methods development to address the nature of the comparator in randomized controlled trials and reverse causality and residual confounding in prospective cohort studies [140,141,143,144,145,146,147]. Recent systematic reviews and meta-analyses have begun to address these important issues. Together with work on potential biological mechanisms of action that underlie any metabolic and endocrine effects of NNSs, they show more consistent signals that support the intended benefits of NNSs in sugars reduction.

### 6.1. Evidence from Randomized Trials

Several systematic reviews and meta-analyses have directly addressed the nature of the comparator issue (caloric versus non-caloric comparators) in randomized controlled trials. These syntheses have examined the ability of NNSs as a class of additives (as opposed to an individual NNS) to displace calories and sugars in substitution for SSBs. The earliest of these syntheses showed that NNSs (especially NNSBs) in substitution for sugars (with caloric displacement) but not water (without caloric displacement) resulted in reductions in energy intake and body weight [148,149,150,151]. These findings are supported by the two largest and most comprehensive syntheses of randomized controlled trials to date that were designed specifically to interrogate the role of the comparator, one commissioned by the Diabetes and Nutrition Study Group (DNSG) for the update of the European Association for the Study of Diabetes (EASD) dietary guidelines [152,153] and the other commissioned by the World Health Organization (WHO) for the development of the new guideline on the use of NNSs [154,155].

**Figure 4:**
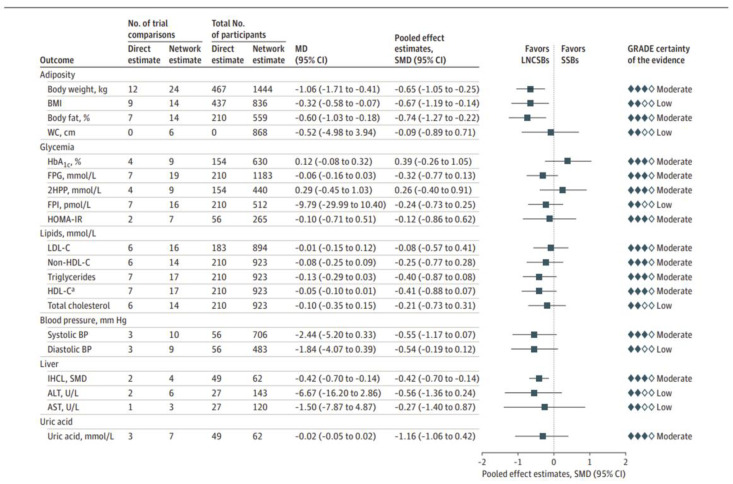
Pooled direct, indirect, and network effect estimates of the effect of the substitution of NNSBs for SSBs (“Intended substitution”) on established intermediate cardiometabolic outcomes. Reproduced from McGlynn et al. [152] under the terms of an open access CC-BY license.

The DNSG-commissioned synthesis [152] assessed three prespecified substitutions of clinical and public health importance: NNSBs for SSBs (the intended substitution with caloric displacement), water for SSBs (the “standard of care” substitution with caloric displacement), and NNSBs for water (the reference substitution without caloric displacement). To increase the information size, network-meta-analyses (as opposed to traditional pairwise meta-analyses) were conducted of 17 randomized controlled trials of the effect of the three prespecified substitutions on 20 established intermediate cardiometabolic outcomes in 1733 adult participants who were predominantly overweight or obese over a median follow-up of 12 weeks (range, 3 to 52 weeks). The substitution of NNSBs for SSBs reduced body weight, BMI, body fat, and liver fat (Figure 4), whereas the substitution of water for SSBs showed non-significant reductions favoring water across the 20 intermediate outcomes. The substitution of NNSBs for water did not show any significant differences except for a greater reduction in body weight and SBP favoring NNSBs and a greater reduction in HbA1c favoring water, suggesting comparable effects of NNSBs and water for SSBs reduction [152].

The WHO commissioned synthesis [154] found similar results, building on an earlier WHO-commissioned systematic review and meta-analysis that had failed to account for the nature of the comparator [138,141]. It assessed the health effects of total food sources of NNSs (especially NNSBs) in substitution for sugars (with caloric displacement) versus water or nothing (without caloric displacement) in 50 randomized controlled trials in adults and children [154]. The substitution of NNSs for sugars (with caloric displacement) reduced caloric intake and downstream body weight and BMI, whereas the substitution of NNSs for water or nothing did not show any differences [154].

New evidence published since the census for these evidence syntheses also confirm the intended benefit of NNSBs. The SWITCH (effectS of non-nutritive sWeetened beverages on appetITe during aCtive weigHt loss) trial [156,157,158], one of the largest and longest randomized trials to date showed that the substitution of NNSBs for other cold drinks as part of a weight loss intervention reduced the caloric intake of sugars and downstream body weight, waist circumference (abdominal fat), LDL-cholesterol, blood pressure, and liver enzymes related to liver fat in 262 overweight or obese participants who completed the trial at 1 year [157].

Taken together, the available evidence from randomized trials supports the use of NNSBs as an alternative to water for replacement of SSBs as part of sugars reduction strategies in overweight/obese adults over the moderate to long term. Several other randomized trials are ongoing and will allow one to assess the nature of the comparator and calories to be displaced by NNSBs and add to this growing line of evidence (ClinicalTrials.gov identifiers, NCT03259685, NCT03944616, and NCT03543644). One of the largest is the Strategies To oppose Sugars with Non-nutritive sweeteners Or Water trial (STOP Sugars NOW), a pragmatic randomized controlled trial of the effect of the replacement of SSBs with NNSBs versus water on changes in glucose tolerance and gut microbiome [159].

### 6.2. Evidence from Prospective Cohort Studies

**Figure 5:**
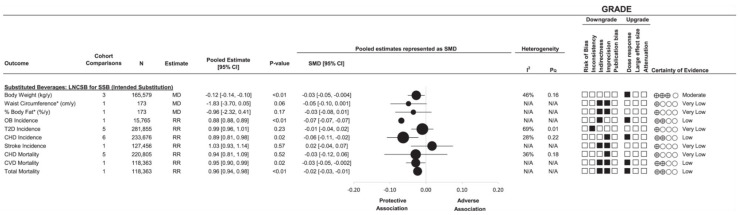
Pooled analyses of estimates of the association of the substitution of NNSBs for SSBs (“Intended substitution”) with clinical cardiometabolic outcomes. Reproduced from Lee et al. [160] with permission from the American Diabetes Association.

There has been considerable methods development in the analysis of observational studies to mitigate the risk of reverse causality and residual confounding. Change-for-change and substitution analyses have been developed which effectively model dietary interventions, providing more reliable and biologically plausible estimates that better align with randomized controlled trials evidence. A second systematic review and meta-analysis commissioned by the Diabetes and Nutrition Study Group (DNSG) for the update of the European Association for the Study of Diabetes (EASD) dietary guidelines [153,160] is the only synthesis to date to use these methods to address the reverse causality and residual confounding in prospective cohort studies. The investigators assessed the association of NNSBs with clinical cardiometabolic outcomes by modeling the exposures as changes in NNSBs intake and substitution effects using the same three prespecified substitutions of clinical and public health importance (NNSBs for SSBs, the intended substitution with caloric displacement; water for SSBs, the “standard of care” substitution with caloric displacement; and NNSBs for water, the reference substitution without caloric displacement). This approach was in alignment with the recent methods developed by the WHO in the assessment of saturated fat and health outcomes to mitigate bias [161]. The investigators identified 14 prospective cohort studies involving 14 cohort comparisons in 416,830 adults that allowed for these analyses. An increase in NNSBs was associated with lower body weight, waist circumference and risk of type 2 diabetes. Similarly, the substitution of NNSBs for SSBs was associated with lower body weight; risk of obesity, CHD, CVD mortality, and total mortality with no adverse associations across other outcomes (Figure 5), whereas the substitution of water for SSBs was associated with lower body weight, waist circumference, and risk of obesity and diabetes and the substitution of NNSBs for water showed null associations [160]. Figure 5. presents the association with the substitution of NNSBs for SSBs (“Intended substitution”) with clinical cardiometabolic outcomes. New analyses of the Nurses’ Health Study and Health Professional Follow-up Study published after the census for these evidence syntheses further reinforce these findings showing the substitution of NNSBs for SSBs is associated with reductions in CVD incidence, CVD mortality, and all-cause mortality in people with type 2 diabetes, a population at high risk for premature cardiovascular disease and death [162]. Unlike the findings from prospective cohort studies using prevalent or baseline analyses [163], these findings align with the higher certainty evidence from randomized trials of intermediate outcomes, supporting the use of NNSBs as an alternative to the standard of care water for the replacement of SSBs in the reduction in prioritized clinical cardiometabolic outcomes.

### 6.3. Potential Mechanisms of Action

#### 6.3.1. “Uncoupling” and “Coupling” Hypotheses

Several mechanisms have focused on the effects of NNSs beyond displacement of added sugars and calories leading to weight loss. The discovery that sweet taste receptors (T1R2/T1R3) are present not just in the oral cavity, but in extra-oral sites such as the intestines, pancreas, heart, and even brain, spurred several hypotheses that NNSs may be interfering in some way with satiety or calorie-sensing by activation of the sweet taste receptor [139,164,165,166,167,168]. The “uncoupling hypothesis” (uncoupling of the sweet taste from the expected calories) [164,165,166] and “coupling hypothesis” (coupling of sweet taste with added calories) [167,168] propose that NNSs alone or through an interaction with calories may lead to disturbed postprandial metabolic or endocrine responses that regulate food intake and glucose metabolism, leading to higher energy intake, weight gain, glucose intolerance, and downstream cardiometabolic risk (the opposite to what has been seen in the randomized trials). To test the biological plausibility of these pathways, a systematic review and network meta-analysis of acute randomized controlled trials was undertaken of the effect different NNSBs (a single matrix) sweetened with single NNS (acesulfame potassium, aspartame, cyclamate, saccharin, stevia, and sucralose) or blends (aspartame + acesulfame potassium; aspartame + acesulfame potassium + cyclamate; acesulfame potassium + sucralose; and aspartame + acesulfame potassium + sucralose) compared with caloric comparators (the intended substitute, SSBs sweetened with glucose, fructose, or sucrose with caloric displacement) and non-caloric comparators (the standard of care, water) on metabolic and endocrine responses related to food intake regulation and glucose metabolism (postprandial glucose, insulin, GLP-1, gastric inhibitory polypeptide (GIP), PYY, ghrelin, leptin, and glucagon) [132]. Three prespecified designs were included: uncoupling (NNSBs consumed alone without added energy or nutrients), coupling (NNSBs consumed together with added calories or nutrients), and delayed coupling (NNSBs consumed as a preload before added energy or nutrients) interventions. The investigators identified 36 trials involving 472 predominately healthy participants. There was no meaningful effect of any NNS alone or as blends on any metabolic or endocrine responses with similar responses to the standard of care water and no differences across NNSs, whereas caloric sweeteners (mainly glucose and sucrose) increased postprandial glucose, insulin, GLP-1, and GIP. Similar patterns were seen across the coupling and delayed coupling designs with a lack of effect of NNS [132]. Specific evidence related to stevia coupled with added calories or nutrients in a biscuit format (coupling design) confirms these findings. A randomized crossover trial from the SWEET consortium showed Stevia Rebaudioside M (StRebM) and neotame in a biscuit format both had lower postprandial glucose and insulin response without changes in other endocrine responses (ghrelin, glucagon-like peptide 1 or pancreatic polypeptide) compared with sucrose in adults with overweight or obesity [169]. In absence of an effect on metabolic and endocrine responses, these findings suggest that any mediation of NNSs on caloric intake, weight change, and downstream cardiometabolic risk factors appears to be through displacement of added sugars and calories.

#### 6.3.2. Microbiome Changes

The role of NNSs in mediating metabolic or endocrine effects through microbiome changes has become an intense focus of interest. The microbiome is an integral part of the body and critical for health, comprising a myriad of types of bacteria. However, normal fluctuations of these bacteria exist in response to many normal foods and other conditions, and how these relate to human health is only recently being explored [170]. Although no conclusions can be drawn yet based on existing data, randomized clinical trials (RCTs) are summarized in this section, as they are leveraged as the gold standard to set clinical guidelines. They predominantly show no effect of extended exposures to an NNS (Table 6).

The first RCT that observed that NNSs may induce dysbiosis in humans and then linked these changes to an impairment in glucose tolerance was published on Nature by Suez and coworkers [171]. This study had one of the highest Almetric scores across all articles in all journals at the time, driving headlines globally (https://www.nature.com/articles/nature13793/metrics, accessed on 17 December 2024). There were, however, multiple sources of bias that limited causal inferences and generalizability. It was uncontrolled (before versus after design with no control group and trial conditions that could have plausibly accounted for the observed differences), underpowered (pilot study with only seven participants), used saccharin at 100% of the acceptable daily intake (ADI) (a minor NNS not used in beverages with a very low prevalence of exposure [171] and relatively hard ADI to reach of 5 mg/kg body weight, equivalent to 45 packets of sweeteners per day for a 60 kg individual [https://www.fda.gov/media/168517/download?attachment, accessed on 17 December 2024]), and achieved statistical significance only through post hoc analyses that arbitrarily classified the seven participants into two groups (four responders, in whom the effect was seen and three non-responders, in whom the effect was not seen). Subsequent studies were designed specifically to address these limitations using double-blind, placebo-controlled, adequately powered randomized controlled designs.

To date, no RCTs have been able to replicate the results using the same 16s RNA sequencing method for assessing microbiome changes and 75g oral glucose tolerance test (75g-OGTT) method (incremental area under the curve) for assessing glucose tolerance in generally healthy participants. The subsequent trials predominantly show no effect of extended exposures to an NNS. Serrano and co-workers [172] showed no effect on the microbiome of saccharin at 400 mg/day (100% ADI) in 46 healthy participants over 2 weeks, while Ahmad and co-workers and Thomson and co-workers [173,174,175] showed no effect on microbiome changes or glucose tolerance of more prevalent NNSs at more real world doses: aspartame at 425 mg/day (14% ADI) or sucralose at 136 mg/day (20% ADI) in 17 healthy participants over 2 weeks [174,175] and sucralose at 780 mg/day (75% ADI) in 34 healthy participants over 7 days [173], respectively. An unblinded (open-label), non-placebo controlled randomized trial also showed no effect of stevia (Steviol glycoside containing drops) on microbiome using the same 16s RNA sequencing method [176] and glucose tolerance using the same 75 g-OGTT methodology [130], compared with usual diet (control) in 27 and 28 healthy participants, respectively, over 12 weeks, although a reduction in energy intake and body weight was seen.

The Weizmann institute group who made the initial observation that NNSs may impair glucose tolerance through inducing dysbiosis in humans [171] is the only group to show an alteration of microbiome by NNSs in a follow-up trial using a double-blind, randomized controlled design with a different analytical approach in [40,177]. Suez and coworkers [177] showed that saccharin (20% ADI) and sucralose (34% ADI) but not aspartame (8% ADI) or stevia as steviol glycosides (75% ADI) induced changes in the microbiome and impaired glucose tolerance in 120 healthy participants over 2 weeks. This study, however, assessed temporal changes in the microbiome using shotgun metagenomics (as opposed to beta-diversity changes using 16s RNA sequencing) and assessed glucose tolerance by a 50g-OGTT using continuous glucose monitoring at home (as opposed to a 75g-OGTT using laboratory measured plasma glucose) [177]. A subsequent double-blind, randomized controlled trial by Kwok et al. [178], which also used shotgun metagenomics to assess microbiome changes, failed to show any effect of stevia as steviol glyosides (25% ADI) on microbiome changes, short fatty acid (SCFA) production, or cardiometabolic risk factors including fasting plasma glucose compared with a sucrose control in 59 healthy participants over 4 weeks, although it was the second trial to show a reduction in body weight (as assessed by BMI).

Robust and reliable findings to support an NNS mechanism involving microbiome changes or mediation of metabolic or endocrine effects through microbiome changes remain lacking and an important research priority. Table 7 presents a summary of LNCSs on microbiome changes. We await the results of the STOP Sugars NOW trial, a pragmatic, randomized controlled trial of the effect of the replacement of SSBs with matched NNSBs (sweetened with the most common NNS blend on the market, aspartame + acesulfame potassium or sucralose alone) versus water on changes in glucose tolerance by 75g OGTT and gut microbiome by 16s RNA sequencing [159].

**Table 6:** Summary of randomized controlled trials assessing the effect of extended intake of different NNS on microbiome changes.

NNS Type	Microbiome Changes in Randomized Controlled Trials					
	Singh et al., 2024 [176]	Kwok et al., 2024 [178]	Suez et al., 2022 [177]	Serrano et al., 2021 [172]	Ahmad et al., 2020 [174]	Thomson et al., 2019 [173]
**Aspartame**			↔		↔	
**Acesulfame Potassium**						
**Sucralose**			↓*		↔	↔
**Saccharin**			↓*	↔		
**Stevia**	↔	↔	↔			

* Significant alteration in microbiome composition seen by trajectory analysis using PERMANOVA, *p* < 0.05. Key: ↔ inNo change; ↓* statistically significant decrease in microbiome diversity.

Key Points:

- The ability of LNCSs to be useful in dietary strategies for reducing intake of calories and sugars in weight management has come under increased scrutiny.
- Systematic reviews and meta-analyses have shown mixed results; however, the preponderance of evidence from randomized trials supports the use of NNSBs are no less effective than water for replacement of SSBs as part of sugar reduction strategies in overweight/obese adults over the moderate to long term.
- More research will be important for improving the certainty of the estimates and clarifying any mechanisms beyond the displacement of added sugars and calories.

## 7. Safety of Low-Calorie and No-Calorie Sweeteners 

### 7.1. Scope of Review

For brevity, this review summarizes the safety of LNCSs permitted for use in the United States.

#### Background: Determination of Food Ingredient Safety and Common Questions Regarding the Safety of LNCSs, as a Class

Authorities such as the United States FDA and the European Food Safety Authority (EFSA) follow strict standards for determining whether a substance is safe for use in food [179,180]. For instance, for substances never previously added to the food supply, research must demonstrate no observable adverse effect with daily intakes very high compared to expected human exposure. An Acceptable Daily Intake (ADI) is then set, which is commonly 100× less than an amount found safe with long term consumption in appropriate animal studies. This provides a wide safety margin for human consumption. Indeed, FDA requires the science supporting the proposed use of a food ingredient to demonstrate “*reasonable certainty* of *no harm*” [181,182]. A slight exceedance above the ADI would also be unlikely to carry significant risk, given the absence of effect found with much higher levels. It is also unlikely that a daily intake notably above the ADI would be regularly consumed. Regulatory authorities consider expected daily intake when deciding to permit a new food ingredient for its intended use.

Types of studies required by regulatory agencies for a new food ingredient range from chemical studies to cellular studies, to high-dose, long-term animal studies and sometimes studies in humans are performed. These are normally to determine if any break-down products (metabolites) are formed in humans following ingestion and how these are dispersed and/or eliminated from the body.

Studies in animals are typically in species that would be exposed to these same metabolites, in addition to the parent compound, at very high levels. Prolonged, high-dose, daily exposure studies allow for exploration of possible effects on not just overall health, but on reproduction, growth and development, organ function and health, life longevity, cancer-causing potential and more. It is also to determine at what point effects might be observed. This helps particularly to gauge the margin of safety. The expected daily intake (EDI) of a proposed new food ingredient is typically below the average daily intake (ADI).

In cases where a substance has already been in the food supply but has not been previously isolated for use in food manufacture, the types of research studies required can be different. This is because some understanding of the safety of the substance may already be known from existing human exposure. For instance, the FDA may not require further research when a substance is “generally recognized as safe” or GRAS, by qualified experts, within the levels expected for use in food.

Regarding any substance we are exposed to, it is important to consider the full body of research when assessing the potential for health effects. Similarly important is the quality of the research conducted for assessing safety. The most common concerns raised about LNCSs are whether they might cause weight gain, cancer or more recently, whether they might impact health by affecting the gut microbiome or be unsafe for children.

Regarding weight gain, the impetus for such claims has largely stemmed from a few academic animal studies and some human observational studies. Numerous high-dose, well-controlled animal studies, required by regulatory agencies, show no evidence of excess weight gain. Observational studies, by their nature, are designed to determine associations between one parameter and another. They are not direct tests for causality of an association. Moreover, experts have acknowledged that associations of overweight in studies looking at LNCS exposure may be the result of reverse causation [183,184].

For example, a study looking at a group of people who have consumed diet soft drinks may find that this consumption is associated with a greater number of persons who are overweight. This association, however, may be a result of overweight individuals choosing a diet soft drink as a part of a calorie intake management strategy, and not the diet soft drink causing weight gain.

Observational studies evaluating effects on body weight also may have several other confounding factors, such as other dietary habits, environmental conditions, etc. A recent paper by certain researchers on behalf of the WHO which concluded low evidence of LNCSs being useful in weight management [137], has also been called to re-evaluate this conclusion because of how dependent it was on observational studies [163]. In contrast to observational studies, randomized controlled trials (RCTs) overall show no evidence LNCSs being able to cause overweight [149,150,151].

Questions about LNCSs, as a group, having an ability to cause cancer may also result from certain observational studies. All LNCSs are generally markedly different on a chemical basis, and observational studies of associations between use of diet soda, for example, cannot help to determine which, if any, LNCSs are related to a finding of increased cancer incidence. As noted above, observational studies are not able to prove causality and may have biasing factors. Even with all this in mind, a recent review of genotoxicity and carcinogenicity research and epidemiological studies concluded that there is no evidence of cancer risk associated with LNCS consumption [185].

As far as the potential for LNCSs to impact health by affecting the gut microbiome, the influence of the gut microbiome on health is still very much an emerging area of research. Concerns about the meaningfulness of LNCSs/gut microbiome research have already been raised [186,187]. The microbiome comprises thousands of species and trillions of microorganismal cells, and the gastrointestinal microbiome is affected by many factors, including age, diet, health status, medication use, and more [170].

Common dietary substances, such as various types of sugars and more complex carbohydrates, such as dietary fiber, as well as dietary fats and certain proteins can all affect changes in the gut microbiome composition [186,188,189,190]. Thus, one must understand if changes observed are within the normal range of changes found with common dietary influences [186]. Additionally, each person’s microbiota composition is relatively unique and can respond differently to different dietary changes [190]. There can also be overlap in the functionality of different types of gut microorganisms. Interpretation of studies can also be confounded by the methods for retrieving and identifying microbiome cells [191]. Moreover, while the microbiome is of clear importance to health, the functional mechanisms that underlie host-microbiome interactions are not clearly known [192].

Finally, in the case of the most popular LNCSs, intakes are incredibly small relative to other nutritional components, so it is difficult to imagine a serious impact on the gut microorganisms to such an extent that there would be major shifts in how these function in our health. Indeed, in contrast to hypotheses of possible adverse effects by interaction with the gut microbiome, the overall research does not support an adverse effect of LNCSs on gut function [193] or overall health, based on numerous regulatory reviews.

Lastly, regulatory agencies look particularly closely for possible effects during growth and development. This is primarily conducted by employing very high doses in surrogate species considered to be appropriate for such evaluations. An important consideration here is how an LNCS is handled by the body (e.g., its absorption, metabolism and excretion profile in animals vs. humans). Also considered is the potential for an LNCS to cross the placental barrier or to enter the milk supply. While some researchers have posed safety questions based solely on the possibility of exposure of fetuses or newborns to an LNCS, this should not be the sole measure of whether a substance is safe. For any substance newly proposed to be allowed in food manufacture, high-dose studies in surrogate species are conducted to evaluate possible effects on such parameters as reproductive performance, neonatal health and development, and health during weaning to adulthood. They also include studies to assess the possibility of birth defects. In general, for an LNCS to be approved, research must support that expected intakes would not pose health risks when used by either pregnant mothers or children.

### 7.2. Biologic Fate and Safety Profile of LNCSs

#### 7.2.1. Acesulfame Potassium (Ace-K)

Acesulfame potassium (acesulfame K or Ace-K) is a potassium salt of 6-methyl-1,2,3-*oxathiazine*-4(3H)-one-2,2-dioxide. Following consumption, it is rapidly and almost completely absorbed into the body [118,194]. Under certain conditions, there is some evidence that the potassium salt can dissociate from acesulfame to yield free potassium; however, there is no clear evidence of this from biological research [118]. FDA and other health authorities report that Ace-K is not metabolized [195,196]. As such, Ace-K provides no calories to the diet. Following absorption, Ace-K is quite rapidly eliminated from the body, primarily in the urine [196].

Ace-K is approximately 200× sweeter than sugar and is heat stable [197], making it suitable for use in a wide variety of foods, including cooked and baked goods [198]. Were there any dissociation of potassium from acesulfame in food systems, the possible contribution of free potassium to the diet from consumption of Ace-K would be relatively small compared to normal dietary potassium intake. For example, assuming complete dissociation of potassium from acesulfame, a 12-ounce can of Ace-K sweetened diet soda would contain about 60 mg potassium, whereas, the average daily intake of potassium from a variety of foods is > 2000 mg day [199].

While providing a sweet taste, some individuals report a bitter aftertaste with Ace-K [200,201], which may be genetically related [202].

Based on safety research conducted in line with regulatory requirements, the FDA has found Ace-K to be safe for use in a wide variety of foods and beverages and assigned to it an ADI of 15 mg/kg/day. It has been used in food manufacturing for over 30 years. Several studies find that intake of Ace-K is generally well below the ADI [203,204,205,206].

#### 7.2.2. Allulose

Allulose is a type of sugar that is metabolized differently from sucrose (common table sugar) and other commonly known sugars, such as glucose and fructose [197]. It exists naturally, but in minute quantities, in certain fruits [207,208], and is sometimes referred to as a “rare sugar.” It is available more widely now, as a result of it being able to be produced by enzymatic conversion of other natural sugars [209,210]. Chemically, allulose is a monosaccharide that is an epimer, or stereoisomer, of fructose [211]. It is also referred to as D-allulose or D-psicose.

Allulose undergoes very limited metabolism and so has very little caloric value—approximately 0.4 cal/g or about 10% of the caloric value of sucrose [212,213]. Most ingested allulose is absorbed from the gastrointestinal tract and most of this is excreted intact in the urine. The fraction of unabsorbed allulose that passes to the large intestine is largely not metabolized and is excreted intact in the feces [214].

FDA lists allulose as “generally recognized as safe” (GRAS) under its intended conditions of use [197]. While absorbed as sugar, there is no evidence of an effect of allulose on blood sugar [215,216]. Some research suggests that a very small amount of allulose may be a substrate for certain gut microorganisms, but overall evidence indicates no significant amount of fermentation. Consistent with this, normal use is not expected to result in gastrointestinal side effects that are sometimes found with excess intakes of poorly digestible substances that can be acted upon by the gut microbiome [217].

Allulose is reported to have a sweetness approximately 70% of the sweetness of sugar [211]. It has good heat stability [218], and so can be used in cooking and baking. Average current intake is estimated to be not more than 200 mg/d [217] which is far lower than amounts found safe in people [216,219].

#### 7.2.3. Aspartame

Aspartame is a dipeptide methyl ester. Following consumption, it is fully and rapidly broken down in the gut to yield phenylalanine and aspartic acid, both of which are naturally occurring amino acids found in many types of protein in the human body and in foods. Aspartame digestion also releases its methyl ester as methanol. Methanol is found naturally in many foods, e.g., fruits, fruit juices, fermented foods and other food types. Both the released amino acids and methanol are absorbed into the body and generally then used for energy or, in the case of the amino acids, for making more protein [220].

Based on safety research conducted in line with regulatory requirements, the FDA first approved aspartame for use in a range of food and beverage categories in 1974, and later as a general-purpose sweetener, under conditions described in its regulation and consistent with good manufacturing processes [221]. Thus, it has been used in food manufacturing for approximately 50 years. It should be noted that aspartame use in cooking and baking can be limited, as it can break down in foods dependent upon time, temperature and pH [222,223].

The breakdown of aspartame can affect a product’s sweetness. The decomposition products of aspartame include those produced in the body following its ingestion, and a few others, primarily diketopiperazine (DKP). No safety concerns have been found with DKP resulting from aspartame intake [224,225].

Aspartame is approximately 200 times sweeter than sucrose (common table sugar). Because of its high sweetness potency, amounts consumed represent virtually no calories. The ADI set by FDA is 50 mg/kg (body weight)/per day. Several studies confirm that aspartame intake rarely exceeds the ADI [203,204,205,226]. This applies to the general population.

However, the FDA notes that persons who have difficulty metabolizing phenylalanine, a result of a rare metabolic disorder called phenylketonuria (PKU), should avoid or restrict aspartame intake. Persons with PKU are normally directed to restrict their intake of foods that may contain phenylalanine (e.g., meats, cheese, and eggs) [227]. Additionally, undetected PKU is unlikely, as testing for PKU in newborns is a common practice.

Controversy over the safety of aspartame has been primarily driven by studies conducted by the Ramazzini Institute (RI), which asserted that aspartame is carcinogenic [228,229]. The RI studies were also the basis for a conclusion of “limited evidence” that aspartame is “possibly carcinogenic to humans” by the International Agency for Research on Cancer (IARC) [230]..

However, multiple well-regarded authorities have found that the RI studies have serious flaws that do not allow for reliable conclusions. The FDA notes that they disagree with the IARC conclusion and, moreover, that the conclusion does not actually mean that aspartame is linked to cancer [197]. EFSA specifically concluded that the RI studies “did not produce any scientific evidence supporting a carcinogenic effect of aspartame” and that “there is no evidence to suggest that aspartame induces cancer according to existing large human population studies” [220].

Overall, Both the FDA and other regulatory agencies have re-affirmed their conclusions that aspartame does not cause cancer and, accordingly, have not changed their assigned ADI for aspartame. A recent systematic assessment of human, animal and mechanistic data also found no evidence for carcinogenicity potential with human consumption of aspartame in foods [231]. Similarly, a review of genotoxicity and carcinogenicity research and epidemiological studies concluded that there is no evidence of cancer risk associated with LNCS consumption [185].

There has also been some attention drawn to aspartame safety in light of the release of methanol following its digestion, but no safety concerns are assigned to this [220,224]. Intake of methanol from aspartame is actually overshadowed by intake resulting from consumption of common fruits such apples and citrus fruits and other dietary sources.

More recently, a publication reported an association of aspartame exposure during pregnancy with increased autism risk in males [232], but others have found that this report is not warranted [159]. Objections include no evidence of a plausible biological mechanism and methodologic issues with the study design. For example, the study utilized dietary recall data, where recalled intake of products that may have contained aspartame included “intake during pregnancies that occurred up to 30 years earlier.” As an observational study, the reported association is also, in any case, not evidence of a causal effect.

#### 7.2.4. Erythritol

Erythritol is a substance in the class of sugar alcohols. It is produced naturally in the human body, to some extent, and is found naturally in plants [233]. Most erythritol is produced commercially by yeast fermentation of simple or complex carbohydrate sources [234,235]. Following consumption in humans, almost all erythritol is absorbed into the body [236,237], but the extent of absorption in humans may be dose-dependent [238]. A small fraction of absorbed erythritol can undergo metabolism to yield erythronate. No safety concerns are noted with this metabolism. Erythritol is then excreted in urine unchanged [236].

Because of its high level of absorption and elimination in the urine, very little of the consumed erythritol reaches the large intestine. Potential metabolism by gut microbiota is therefore limited. Indeed, some studies show no metabolism of erythritol in humans [233]. It is known that gut microbiota can feed on certain sugar alcohols, resulting in the production of intestinal gas and, with excessive intakes, a laxative effect [239,240,241]. Erythritol, however, has been shown to be significantly better tolerated than other sugar alcohols, and typical use can normally be expected to avoid the gastrointestinal reactions sometimes found with other sugar alcohols [241,242]. The FDA lists erythritol as “generally recognized as safe” [243]. Excessive intakes cannot, however, be excluded from causing a laxative effect [236].

While a small proportion of consumed erythritol may be metabolized by gut microbiota, the likelihood of this providing meaningful calories to the body is low. Its nutritive value is estimated to be <0.4 kcal/gm [244,245], and, for the purposes of nutritional labeling, erythritol is assumed to contain 0 kcal/gm [246,247].

Erythritol sweetness is reported to be, on average, about 30% as sweet as sugar [233], ranging from 50% to 80% as sweet, depending on the concentration tested [248,249]. Its sweet taste can also be accompanied by a cooling taste effect [250]. Erythritol will not break down under heating conditions typical with food manufacture and is used in a wide variety of products [251].

While in the class of sugar alcohols, erythritol is not a sugar and does not yield sugar with consumption and, consistent with this, has no effect on blood glucose levels [236,252,253,254].

#### 7.2.5. Mogrosides

Mogrosides are sweet substances found in Monk fruit, or Swingle fruit, also known as *lo han guo*, which is native to southern China. Monk fruit has been cultivated there for centuries for consumption and use in medicinal teas and other traditional medicines [255,256]. No adverse effects on human health or development have been reported with these uses [257].

Chemically, mogrosides are a type of cucurbitane triterpenoid saponin [258]. There are numerous types of mogrosides present in the fruit that are all chemically similar: each has a mogrol base that has a varying level of glycosylation (attached glucose or other sugar molecules) [259,260]. The different types of mogrosides are typically denoted by different (roman) numerical suffixes. Mogroside V, for example, is commonly the most predominant type of mogroside in commercial monk fruit extracts [257,261].

Research in animals shows that there is very limited systemic absorption of mogrosides and that absorbed mogroside(s) are largely, if not entirely, non-metabolized and ultimately excreted in the urine. Unabsorbed mogrosides are acted upon by gut microorganisms, which cleave from the mogrol backbone its attached glucose units. Full de-glycosylation leaves free mogrol, which also has limited absorption [121,262,263]. Consistent with the presence of different mogrosides in monk fruit extracts, and with varying levels of deglycosylation possible, in addition to other breakdown products through interaction with intestinal microorganisms, a wide range of mogroside metabolites have been detected [264,265].

The metabolic fate of mogrosides in humans is generally expected to be the same as what has been found with laboratory animals. Human intestinal fecal homogenates, which harbor active gut microbiota, show similar de-glycosylation of mogrosides as to that found in animals [121].

The FDA lists several monk fruit extracts with high concentration of mogrosides as GRAS [266]. The GRAS Notices for these extracts include consideration of historical uses, metabolic and toxicologic research, and expected intakes.

Studies show that the sweetness potency of different mogrosides is related to the number and stereoconfiguration of the glucose groups present in the molecule [267,268]. Typical monk fruit extracts have a sweetness about 100–250 times sweeter than sugar. A bitter after-taste has also sometimes been reported with certain monk fruit extracts/mogrosides [269,270]. As such, when used by food manufacturers, monk fruit extracts may be blended with another LNCS to achieve a desired sweetness profile. Mogroside fruit extracts retain their sweetness stability in typical food manufacturing conditions, and can be used to sweeten a variety of liquid products and foods [271,272], including baked goods [273,274].

In combination with their low level of absorption and sweetness intensity, mogrosides can be considered to provide no calories to the diet. As a relatively new LNCSs to countries outside of those where it has long been in use, average daily mogroside intake can be expected to be low, particularly given that its use may be more commonly in combination with other sweeteners.

Some health benefits have been implicated with mogroside use [3], however, much more research is needed to evaluate the likelihood of beneficial effect with ordinary consumption [275].

#### 7.2.6. Neotame

Neotame is a dipeptide methyl ester derivative, synthesized from aspartame by reductive alkylation [276]. It is more heat-stable than aspartame, owing to the differences in its structure, and can be used in cooking and baking, under expected use conditions [277,278]. Neotame is also about 10,000x sweeter than sugar, by weight [197,279].

Research indicates that most consumed neotame will be absorbed into the human body and, following this, undergoes de-esterification, which releases methanol. The amount of methanol produced is far less than what is expected to be consumed from other dietary sources, and so represents no safety concern.

The body further metabolizes neotame resulting in the release of phenylalanine and other metabolites, which have also been found to represent no safety concern, based on the collective research. Possible exposure to phenylalanine, an amino acid also released with the digestion of proteins commonly in the diet, is considered to be so low as to be inconsequential, including for persons with PKU [278,280]

While neotame is metabolized, it is effectively calorie-free. This is because so little can be expected to be consumed, as a result of its extreme sweetening potency.

Based on safety research conducted in line with regulatory requirements, the FDA has found neotame to be safe for use as a general-purpose sweetener within the conditions provided in its regulation [280]. The ADI set by the FDA for neotame is 0.3 mg/kg (body weight)/day [197]. Actual intakes of neotame have been found to be below the ADI [281].

#### 7.2.7. Saccharin

Saccharin is a benzoic sulfimide. When added to food, it is typically as its sodium salt, since its acid form is far less soluble [282]. The calcium salt of saccharin is also available for use and typically of more interest to persons wishing to restrict their sodium intake. In foods, saccharin salts dissociate to yield free saccharin and their salts.

Following consumption, saccharin is absorbed into the body, intact, and is excreted unchanged, primarily in the urine [118,283]. Accordingly, saccharin is non-caloric.

Saccharin is about 200–700 times sweeter than sugar. While providing sweetness, a bitter aftertaste may be detected by some individuals, which has been reported to be genetically related [201,284,285].

Based on safety research and long historical use, saccharin is permitted for use in a wide range of products, and can be used in cooking and baking [197,286]. It has generally good thermal stability [287,288,289].

The ADI set by FDA is 15 mg/kg (body weight)/per day [197]. Several studies find that daily saccharin intake for the general population is well below the ADI [203,205,226,290].

While saccharin was once thought to be a possible carcinogen, specifically because of an increased risk of bladder cancer found in rats consuming extreme amounts of saccharin daily, a wide body of research has established that the results found in these rats are not relevant to humans [197,291]. Saccharin is now considered non-carcinogenic by regulatory agencies around the world. A recent meta-analysis of the current literature similarly concludes that saccharin (is not a cause for concern in risk of bladder cancer [292].

There has also been some concern voiced over the safety of saccharin use during pregnancy. This was based on a study in monkeys where a single dose of radioactive saccharin was administered intravenously to pregnant monkeys [293]. That study reported that saccharin might accumulate in the fetus with repeated maternal exposure.

However, a two-generation study where pregnant rats were fed a diet containing 5% saccharin showed that the fetus does not accumulate saccharin with repeated maternal intake [294]. Additionally, animals exposed to daily saccharin intakes of up to 100 to 400 times the human ADI do not suggest risk of malformations [295], and there was no increased risk of spontaneous abortions found in a case control study of women who consumed saccharin [296].

A meta-analysis of studies in women consuming LNCSs also found no linear dose–risk relation in incidence of preterm deliveries in women reporting intake of LNCSs [297]. In general, the overall data support that LNCSs, including saccharin, can be safely used during pregnancy [298].

#### 7.2.8. Steviol Glycosides

Steviol glycosides are sweet substances found in the leaves of the stevia plant (*Stevia rebaudiana* Bertoni). Stevia leaf extracts have been used for hundreds of years in Latin America, in countries where the plant natively grows [299]. No adverse effects on human health or development have been reported with these uses. Chemically, steviol glycosides are in the class of diterpene glycosides [300]. There are a wide range of steviol glycosides in the stevia leaves, which are all chemically similar: each has a diterpene steviol base that has a varying level of glycosylation (attached glucose or other sugar molecules) [118].

Steviol glycosides are poorly, or not at all, absorbed. They are also not digested in the upper gastrointestinal tract, however, with transit to the lower intestine, gut microbiota cleave off attached sugar residues, leaving free steviol, which is absorbed systemically. Absorbed steviol undergoes glucuronidation in the body and the resulting steviol glucuronide is excreted via the urine in humans [118,301]. Glucuronidation is a common biological process that aids in excretion of xenobiotics [302].

High-purity (>95%) steviol glycosides have been deemed GRAS by the FDA. Consumers should be aware that this GRAS determination pertains specifically to the proposed use of high-purity steviol glycoside sweeteners, and does not extend to the use (of crude stevia leaf extracts or intact stevia leaves [197]. Such uses cannot rule out the presence of other substances that might have other effects.

Steviol glycosides have a sweetness potency of about 180–350 times that of sucrose [303]. The sweetness intensity is known to vary dependent on the number and type of sugar residues on the steviol backbone and the position of attachment [304].

Since it is not metabolized for energy following any possible absorption, steviol glycosides provide no calories to the diet. Steviol glycosides are heat stable [305] and so can be used in the manufacture of foods required to be heated, such as baked goods.

Several studies confirm that average daily intake level is well within levels expected to be safe [204,226,306,307].

#### 7.2.9. Sucralose

Sucralose is a disaccharide with chlorine substitutions in place of certain hydroxyl groups. The chlorine substitutions prevent sucralose from being able to be digested or used by the body as a source of energy. As a consequence, sucralose has no calories.

Radiolabel studies show that most consumed sucralose, about 85%, is not absorbed into the body, and that the unabsorbed fraction is excreted unchanged in the feces. This provides significant evidence that sucralose is not a substrate for gut microorganisms. Of the ~15% that is absorbed, most of this is also not changed in the body. A portion undergoes glucuronidation—a common xenobiotic biological processing that results in the addition of glucuronic acid(s) (glucose with an acid group, found widely in nature) to the target molecule.

The addition of glucuronide can make substances more water-soluble, and thus can be an aid in ensuring their excretion via the urine. Absorbed sucralose and its glucuronide conjugates are excreted quite rapidly in urine. While sucralose contains chlorine, no free chlorine is released from sucralose to the body, as it is not broken down in the body for energy, nor is sucralose broken down to yield smaller chlorinated molecules [118,308].

Similarly, consistent with its lack of digestion in the body, overall research shows that sucralose has no effect on blood glucose levels or regulation [309]. In general, it is worth noting that disaccharides and their derivatives that are not broken down in the gastrointestinal tract are also known to be poorly absorbed, and when present systemically, to be largely excreted unchanged [310,311,312,313].

Sucralose is about 600 times sweeter than sugar. Based on safety research conducted in line with regulatory requirements, the FDA has generally found sucralose to be safe for use in foods and beverages. Since it is heat stable, it can also be used in cooking and baking [314,315,316].

The ADI set by the FDA is 5 mg/kg/day. Several studies confirm that sucralose intake rarely exceeds the ADI [203,204,205,226,281,317,318].

A few studies have hypothesized a potential breakdown of sucralose with its use in cooking and baking; however, these studies were found to be performed under abusive conditions or other conditions wholly unrealistic for the expected manufacture of foods [319]. Importantly, breakdown products hypothesized to form under normal cooking/baking conditions were not found in a recent study of the manufacture of different sucralose-sweetened food products, such as cake, cookies and pizza (sauce made with sucralose and used in pizza) [319]. An earlier study using radiolabeled sucralose in the preparation of a variety of baked goods also showed no evidence of sucralose breakdown [314].

A study by the Ramazzini Institute (RI) [320] had previously roused some public concern for the possibility that sucralose might be able to cause cancer. However, this study, and other sweetener studies conducted by the RI, have been found to be unreliable for assessing the potential for carcinogenity, based on serious flaws in their methodology [220,321,322,323,324]. Additionally, a plausible mechanism for sucralose causing cancer is not supported by research [321,325]. A recent review of genotoxicity and carcinogenicity research and epidemiological studies, also concludes that there is no evidence of cancer risk associated with LNCSs (including sucralose) consumption [185].

#### 7.2.10. Tagatose

Tagatose, or d-tagatose, is a type of sugar that is metabolized differently from both sucrose (common table sugar) and other commonly occurring sugars, such as milk sugar, or lactose [197]. Chemically, tagatose is a stereoisomer of d-fructose and an isomer of d-galactose [326,327], both of which are sugars that result from digestion of lactose. Tagatose is considered a “rare sugar”, since it is found in a very limited number of natural sources and in minute quantities [328].

However, it can be formed during heat-treatment of milk [329] and with milk fermentation [330], so it can often be found in dairy products [331]. It is available more widely now, by large-scale enzymatic conversion of lactose or galactose or other sugar-derivatives [328,332].

Research indicates that only about 15–20% of ingested tagatose is absorbed from the small intestine, which is then broken down in the body following a metabolic pathway identical to that of fructose [333,334]. Consistent with its poor absorption, tagatose has a much lower caloric value compared to sugar: about 1.5 cal/g or about 20–25% of the caloric value of sucrose [335].

Unabsorbed tagatose passes through to the large intestine where it is fermented by indigenous microorganisms to yield products commonly found with gut microbial fermentation of dietary fiber, e.g., short chain fatty acids and certain gases [334,336,337,338,339].

In 2002, safety studies were reported to be conducted following the recommendations in the FDA “Red Book” [331]. FDA has since listed tagatose as GRAS, under its intended conditions of use [197]. Some gastrointestinal symptoms may result with a tagatose intake of ~30 g, or possibly less in sensitive individuals [340,341,342]. These are typically reasonably well-tolerated and transient and have been found to occur with larger intakes of other poorly absorbed substances. They are considered to result from microbial fermentation of such substances in the large intestine [239,343].

It should be noted that tagatose consumption is advised against for persons with hereditary fructose intolerance [344]. In this rare condition, fructose cannot be metabolized by the body, so it is expected that tagatose would also not be able to be metabolized.

Tagatose has a sweetness potency slightly less (~10% less) than sugar [248]. It has good sweetness stability in normal food processing [345]. While some degradation can occur with prolonged heating [346] this is consistent with a maillard reaction that results in browning [347], which is similarly found with prolonged heating of sucrose.

#### 7.2.11. Thaumatin

Thaumatin is the name given to a group of proteins (e.g., Thaumatin I, Thaumatin 2) found in the West African Katemfe fruit (*Thaumatococcus danielli*) [348,349], extracts of which have been used for hundreds of years to help provide sweetness to certain beverages [350]. Thaumatin-comprising proteins are relatively small proteins and they are very similar, chemically: each has a single polypeptide base of 207 amino acids [351,352,353]. Like with ordinary dietary proteins, thaumatin is readily digested to yield its amino acid components, which are absorbed into the body [354,355]. Thaumatin proteins are intensely sweet—approximately 2000 times sweeter than sugar—so only a minute amount will confer a sweetness desirable for food palatability. Based on safety research, including its biologic fate, the FDA lists thaumatin as GRAS for its intended uses in food [197,356]. While some proteins can be allergenic, thaumatin use in foods is considered unlikely to be allergenic, based on safety research in both humans and animals and also given its rapid digestion [355]. Further, due to its sweetness, only minute quantities would ever be expected to be ingested with expected intakes.

Thaumatin’s sweet taste has been reported as more lingering, compared to some other sweeteners [357,358]. For this reason, it is often blended with other sweeteners to achieve a desired sweetness profile in a finished good. On the other hand, thaumatin is also known to be useful in masking bitter tastes [359,360] and improving other flavors [361] and is permitted for use, and listed as GRAS, as a flavor enhancer [362,363].

Thaumatin can be used in cooking and baking in most food production scenarios. It can lose sweetness under certain conditions. For example, as normally occurs with other dietary proteins, prolonged high heat can cause thaumatin proteins to degrade. Exposure to basic conditions (pH > 7), not typically encountered in foods, can also cause thaumatin proteins to aggregate, or cluster, which can affect the way they can interact with the sweet taste receptor and lead to loss of sweetness [364,365].

Key Points:

- The FDA and other regulatory bodies follow rigorous standards in the evaluation of proposed new food ingredients, including LNCSs.
- A wide body of research supports the idea that approved LNCSs are safe for use.
- Over-reaching conclusions drawn from limited and/or unreliable research has led to the most common concerns raised about the safety of LNCSs.

## 8. Implications—Gaps in the Evidence and Recommendations for Further Research

With growing health concerns and a global focus on reducing sugar consumption, the food and beverage industry has experienced a shift towards LNCSs as options for sugar. The increasing prevalence of LNCSs in the food supply presents challenges and knowledge gaps exist.

First, consumers struggle to understand and differentiate between the types of LNCSs and their definitions as well specific attributes. Compounding the lack of knowledge are the inconsistent dietary consumption guidelines and reduction approaches for sugar intake. Little scientific evidence exists about how the categories of total sugars, added sugars and free sugars were defined, named, and assigned, with consumer communications positioning defined and determined. When substituting LNCSs for sugar, consumers desire an undetectable difference in taste between sweeteners and knowing how to effectively use them in their diets.

Further, the inconsistent presence of standardized FOP labeling for products containing sugar creates difficulties for consumers in making informed choices when choosing food and beverages. Additionally, incomplete knowledge of taste and sensory perception related to LNCSs contributes to potential underutilization of sweeteners, as some consumers may find their taste profiles less appealing than sugar.

Gaps in research on the long-term metabolic efficacy and impact of LNCSs. While short-term studies have shown promising results, the scientific community lacks comprehensive data on the extended effects of LNCS consumption on human health. To better understand the efficacy and effectiveness of LNCSs on obesity, diabetes, and the microbiome, needed are more randomized, controlled, clinical trials.

To address the totality of the issues, establishing clear, evidence-based guidelines for LNCS consumption and sugar reduction is crucial. In the case of sugar and LNCSs, confusion may persist when a product category contains FOP labels warning against the use of both. To improve consumer understanding at the point of purchase, creating standardized FOP labeling systems for products containing LNCSs is essential. This would allow for easier comparison between products and help consumers identify products with LNCSs.

As it relates to advancing the science of LNCSs, two potential areas of investigation present future opportunities. First, additional studies would advance insights about improving the taste and sensory properties of LNCSs to enhance their appeal and increase adoption. Second, long-term studies on the metabolic effects and overall safety of LNCSs use will help to cement our understanding of their impact on human health.

Finally, collaboration between health organizations, policy makers, program developers and the food industry to ensure consistency and wide-scale adoption would aid in shared application and understanding of sweeteners for the public’s health and wellness. Shared interests in this space serve to drive innovation in LNCS development, ensuring these sweeteners continue to evolve to meet both health, safety, and taste requirements.

## 9. Conclusions

The growing global concern over the detrimental health consequences of excessive sugar consumption has catalyzed a shift towards a more mindful approach to health and wellness among public health organizations, policymakers, regulatory bodies, and consumers alike. In response, food and beverage companies have taken measures to innovate and reformulate their product portfolios, adding LNCSs as viable alternatives to sugar.

The greater sweetness intensity of LNCSs compared to sucrose allows for the use of lesser amounts to achieve a similar level of sweetness, facilitating a reduction in an individual’s caloric and sugar consumption. Furthermore, the substitution of LNCSs for sugar supports individual and public health outcomes by addressing issues related to obesity, diabetes, and chronic illnesses.

Overall, large, comprehensive systematic reviews and meta-analyses showed that the intended substitution of NNSs for added sugars (especially NNSBs for SSBs) reduces body weight and downstream weight-related cardiometabolic risk factors in randomized controlled trials. The substitution is associated with reductions in incident obesity and coronary heart disease, cardiovascular mortality, and total mortality in prospective cohort studies. In addition, a few randomized controlled trials are starting to address the impacts of LNCSs on gut microbiome and has not shown any detrimental effects. Lastly, emerging evidence from in vitro and a randomized controlled trial have investigated food intake and satiety management and suggests that natural LNCSs may be beneficial.

The scientific literature presented about product segmentation; dietary consumption and reduction guidance; front-of-package labeling, taste and sensory perception and physiology; metabolic efficacy and impact; and overall safety of reinforce the viability of LNCSs as options for individuals seeking to attain their health and wellness goals. The diverse range of LNCSs available in global food and beverage choices, coupled with their varying sweetness intensities, offers enjoyment and pleasure to consumers on their respective health and wellness journeys.

## Figures and Tables

**Table 1:** Dietary sugar consumption guidance terms and definitions.

Issuing Authority	Term and Definition
World Health Organization (WHO) [10,11]	Free Sugars All mono- and disaccharides except those naturally occurring in fruit, vegetables or dairy. This also includes all sugars during processing and preparation as well as sugars naturally present in juice or pureed fruit and vegetables
European Food Safety Authority (EFSA) [14]	Added and Free Sugars Free sugars include added sugars plus those naturally present in honey and syrups, as well as in fruit and vegetable juices and juice concentrates.
United States Department of Agriculture (USDA) [13]	Added Sugars Added sugars are sugars and syrups that are added when foods or beverages are processed or prepared. This does not include naturally occurring sugars such as those in milk and fruits. Added sugars provide calories without providing additional nutrients.
United States Food and Drug Administration (FDA) [15]	Total Sugars include sugars naturally present in many nutritious foods and beverages, such as sugar in milk and fruits as well as any added sugars that may be present in the product. There is no daily value * for total sugars because no recommendation has been made for the total amount to eat in a day. Added sugars include sugars that are added during the processing of foods (such as sucrose or dextrose), foods packaged as sweeteners (such as table sugar), sugars from syrups and honey, and sugars from concentrated fruit or vegetable juices. They do not include naturally occurring sugars that are found in milk, fruits, and vegetables.

* The Daily Values are reference amounts (in grams, milligrams, or micrograms) of nutrients to consume or not to exceed each day.

**Table 2:** Global sugar guidelines consumption overview.

Geographic Region/ Organization	Country SSB Tax in Effect *	Sugar Intake Recommendation (Yes/No)	Qualitative Recommendation	Quantitative Recommendation
WHO [11]		Yes	For both adults and children, free sugars (2015): <10% of total energy intake (50 g or 12 tsp for 2000 kcal/day) <5% would provide additional health benefits	For both adults and children, free sugars (2015): <10% of total energy intake (50 g or 12 tsp for 2000 kcal/day) <5% would provide additional health benefits
EFSA [26]		Yes	An upper level or a safe level of intake could not be set. Based on available data and related uncertainties, the intake of added and free sugars should be as low as possible in the context of a nutritionally adequate diet. This opinion can assist EU Member States in setting national goals/recommendations	NA
Asia	India * [16]	Yes	Minimize the use of processed foods rich in salt, sugar, and fats. For prevention of diet-related chronic diseases, sugars and refined cereals should be sparingly used	A portion: 5 g sugar Adults: 4 portions per day for sedentary people 6 portions per day for people with moderate activity 9–11 portions per day for people with heavy activity Infants: 2 portions per day Ages 1–9: 3–4 portions per day: Ages 10–18: 4–6 portions per day
	China [17]	Yes	NA	For both adult and children, added sugars: <50 g/day Ideally < 25 g/day
	Indonesia [27]	Yes	Limit consumption of sweet, salty and fatty foods	Sugar recommendations according to energy adequacy for aged groups: 40 g: for pregnant and breastfeeding women, all ages (except men 50–64 y). 20 g: for men 50–64 years.
	Pakistan *	No	NA	NA
	Bangladesh * [19]	Yes	Take less sugar, sweets or sweetened drinks	Range of population free sugars intake goal: <10% total energy Consume not more than 25 g (5 teaspoons) of sugar per day.
AustraliaOceania	Australia [20]	Yes	Limit intake of foods and drinks containing added sugars such as confectionary, sugar-sweetened soft drinks and cordials, fruit drinks, vitamin waters, energy and sports drinks.	NA
	Papua New Guinea	No	NA	NA
	New Zealand [21]	Yes	(2020) Adults: Choose or prepare foods and drinks with little or no added sugars (2012) Children (aged 2–18 y): Choose or prepare foods and drinks with little or no added sugars. Limit the offer of high fat, sugars and salt (HFSS) foods and drinks. (2021) Baby and toddler (<2 y): when preparing food for your baby or toddler, do not add salt or sugars. If using commercially prepared foods, choose those that are low in salt and with no added sugars. (2013) Older people: Prepare foods or choose pre-prepared foods, drinks and snacks with little added sugars (limit your intake of high-sugars foods).	NA
Europe	Russia	No	NA	NA
	Germany [22]	No	For a general healthy population: Reduce sugar and salt intake.	NA
	United Kingdom * [23]	Yes	Have some dairy or dairy alternatives (such as soya drinks); choosing lower fat and lower sugars options. If consuming foods and drinks high in fat, salt or sugars, have these less often and in small amounts.	No more than 5% of the energy we consume should come from free sugars. Ages ≤ 1 y: NA 2–3 y: M 15 g, F 13 g 4–6 y: ≤19 g/d 7–10 y: ≤24 g/d ≥11 y: ≤30 g/d
	France * [24]	Yes	Fruit juice is very high in sugar and low in fiber. If you drink this, the recommendation is to consume no more than one glass per day and to favor pressed fruit. The recommendation is to limit sugary drinks, fatty, sugary, salty and ultra-processed foods.	NA
	Italy * [25]	Yes	Sugars, sweets and sugars sweetened beverages: less is better	Total sugars: ≤15% total energy; Free sugars: ≤10% total energy (Guide: 25 g sugars correspond to about 5% of the energy for a 2000 kcal/day diet)
North America	United States [13]	Yes	Limit foods and beverages higher in added sugars, saturated fat, and sodium, and limit alcoholic beverages.	Adult (>2 y): Added sugars: <10% total calories <2 y: avoid foods and beverages with added sugars.
	Mexico * [28]	Yes	Drink plain aguas frescas or flavored water without added sugars instead of sweetened drinks such as soft drinks, juices and aguas frescas.	Maximum suggested sugars consumption per day: <6 months: avoid added sugars; 2–5 y: 1–2 servings; 6–12 y: 2 servings; 13–18 y: 2–4 servings; ≥19 y: 2 servings. (1 serving: 2 teaspoons, 1/3 cup, or 1/4 can)
	Canada [29]	Yes	NA	Free sugars: <10% of total energy intake
South America	Brazil * [30,31]	Yes	Use oils, fats, salt, and sugars in small amounts when seasoning and cooking natural or minimally processed foods and to create culinary preparations.	NA
			Do not offer sugars or preparations or products which contain sugars to children until 2 years of age;	NA
	Colombia [32,33]	Yes	≥2 y: To maintain a healthy weight, reduce the consumption of packaged products, fast foods, soft drinks and sweetened drinks.	NA
			<2 y: Do not offer your child canned milk, commercial compotes, boxed baby cereals, packaged products, deli meats, fast foods and sugary drinks. Pregnant women: For your health and that of your baby, avoid fast foods, packaged products, sodas, sugary and energy drinks.	NA
	Argentina * [34]	Yes	Limit the consumption of sugary drinks and foods high in fats, sugars and salt.	Free sugars: <10% of total energy intake
	Peru * [35]	Yes	Protect your health, avoid weight gain by reducing the consumption of added sugars in your meals and drinks.	NA
	Venezuela [36]	No	NA	NA
Africa	Nigeria * [37]	Yes	Decrease consumption of sugars and food high in sugars content Children (25–60 months): Limit the consumption of sugary food School-aged children (6–11 years): Encourage consumption of good quality snacks but limit the consumption of sugary snacks Adults (male and female): Limit intake of salt, bouillon cubes and sugars.	NA
	Ethiopia * [38]	Yes	Limiting the addition of salt and sugars in foods and drinks, including coffee Limit the use of sugars, sweets and sugary soft drinks	Limit intake of sugars, sweets, and soft drinks to below 30 g per day Added sugars and sugars-sweetened beverages: recommend 15 g for all ages Limit intake of sugars, sweets, and soft drinks to below 30 g per day Added sugars and sugar-sweetened beverages: recommend 15 g for all ages.
	Egypt *	No	NA	NA
	Democratic Republic of the Congo *	No	NA	NA

* Country-level taxes on sugar-sweetened beverages (SSBs) in effect.

**Table 3:** LNCS consumption position guidance for persons with diabetes.

Issuing Authority	LNCS Position
American Diabetes Association [39]	“Counsel people with prediabetes and diabetes that water is recommended over nutritive and nonnutritive sweetened beverages. However, the use of nonnutritive sweeteners as a replacement of sugar-sweetened products in moderation is acceptable if it reduces overall calorie and carbohydrate intake”.
Diabetes Australia [40]	“The use of alternative sweeteners could assist in maintaining the palatability of foods and beverages with the absence of sugar and with less energy (kJ)”. “Non-nutritive sweeteners include aspartame, sucralose and stevia. These do not influence blood glucose levels and may be a useful alternative for replacing added sugar”.
Diabetes Canada [41]	“Limit intake of free sugars to less than 10% of total daily calorie (energy) intake. This is approximately 50 g (12 teaspoons) of free sugars consumption per day based on a 2000-calorie diet”. “Limit intake of sugar sweetened beverages (SSB) and drink water in their place”. “Promote the intake of whole foods and reduce the intake of free sugars throughout life for overall health”. “Low calorie sweeteners are one tool available for sugar intake reduction efforts”.
Diabetes UK [42]	“LNCS can be used as a ‘stepping stone’ to reduce intake of sugar in the diet as a part of an overall healthy eating plan”. “LNCS are shown to be safe, and they can be used as part of a strategy for adults and children in the management of weight and diabetes. LNCS sweetened beverages may be helpful when they are used as a substitute by regular consumers of sugar-sweetened beverages and as long as substitution doesn’t lead to later compensation with increased energy intake. This approach may be helpful for people who are accustomed to a sweet taste and for whom water, at least initially, is an undesirable option”.

**Table 4:** Current FOP labeling landscape.

Mandatory Policies		
Canada [68] To be implemented 1 January 2026	A black and white nutrition symbol. It has a magnifying glass and highlights what the food is high in: sodium, sugars, saturated fat or any combination of these.	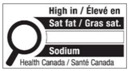
Mexico [69]	Black and white octagon warning labels for each excess nutrient in the product: calories, sugars, saturated fats, trans fats, sodium. Additional warning labels for caffeine and sweeteners to be avoided in children.	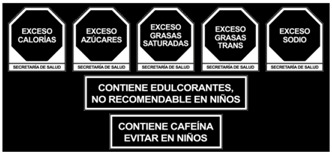
Argentina [70,71]	Black and white octagon warning labels for each excess nutrient contained in the product: calories, sugars, saturated fats, total fat, sodium. Additional warning labels for caffeine and sweeteners to be avoided in children (not pictured).	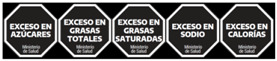
Bolivia [72] Not yet implemented	Traffic light system that uses red, amber and green colors to indicate high, moderate and low levels of saturated fats, added sugars and sodium.	(not available)
Brazil [73]	Black and white warning labels; It has a magnifying glass and boxes for what the food is high in: added sugars, saturated fat and/or sodium.	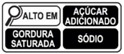
Chile [74]	Black and white octagon warning labels for each nutrient in high amounts found in the product: calories, sugars, saturated fats, and sodium.	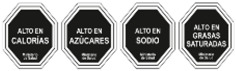
Colombia [75]	Black and white octagon warning labels for each excess nutrient contained in the product: sugars, saturated fats, total fat, and sodium. Additional octagon when sweetener is used.	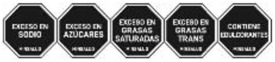
Ecuador [76]	Traffic light system that uses red, amber, and green colors to indicate high, medium, and low levels of fat, sugars and salt. Different sized bars reflect the concentration of these nutrients.	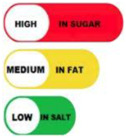
Peru [77]	Black and white octagon warning labels for each nutrient in high amounts found in the product: sugars, saturated fats, and sodium. Additional octagon when product contains any amount of trans fat.	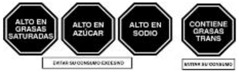
Venezuela [78,79] To be fully implemented December 2024	Black and white octagon warning labels for each nutrient in high amounts found in the product: sugars, saturated fats, trans fats, and sodium (not pictured).	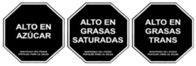
Uruguay [71,80]	Black and white octagon warning labels for each excess nutrient contained in the product: total fat, saturated fats, sodium, and sugars.	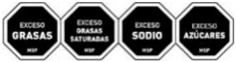
Iran [81]	Traffic light label that uses red, amber and green colors to indicate high, moderate and low levels of sugars, fat, salt, and trans fatty acid.	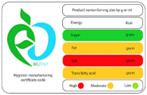
Israel [82]	Red symbols for each nutrient in high amounts found in the product: sugars, salt, and saturated fats.	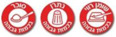
Sri Lanka [83,84]	Traffic light label for beverages that uses red, amber and green to denote levels of sugars in the product.	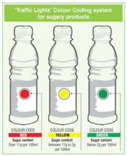
Singapore [85]	Nutrition grading system for beverages that uses a four-point, color coded scale based on sugars and saturated fat levels.	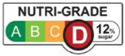
Thailand [86]	Guideline Daily Amount (GDA) monochrome label that gives values of energy, sugars, fat, and sodium in a product.	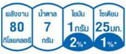
Government-supported Voluntary Policies		
Australia and New Zealand [87]	Summary score system that calculates an overall rating based on the nutritional profile of a product and presents in the form of stars.	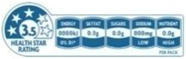
Austria, Belgium, France, Germany, Luxembourg, Netherlands, Portugal, Spain, and Switzerland [88]	Nutrition grading system that uses a five-point color -coded scale that asses a product’s nutritional value.	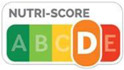
Brunei [89]	Positive endorsement system that identifies a healthier choice by using a red seal with checkmark based on the nutritional profile of a product.	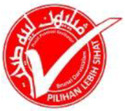
China [90]	Positive endorsement system that identifies a “smart choice” or “healthier choice” (not pictured by using a seal with checkmark based on the nutritional profile of a product.	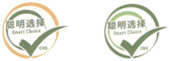
Croatia [91]	Positive endorsement system that identifies a healthier choice by using a green logo based on the nutritional profile of a product.	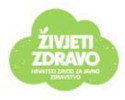
Czech Republic, Poland [88]	Positive endorsement system that identifies a healthier choice by using a blue logo with checkmark based on the nutritional profile of a product.	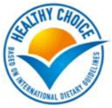
Denmark, Iceland, Lithuania, Norway, and Sweden [88]	Positive endorsement system that identifies a healthier choice by using a green keyhole logo based on the nutritional profile of a product.	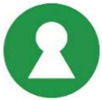
Finland [88]	Positive endorsement system that identifies a healthier choice by using a heart symbol logo based on the nutritional profile of a product.	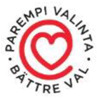
Indonesia [92]	Positive endorsement system that identifies a healthier choice by using a green checkmark logo based on the nutritional profile of a product.	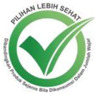
Israel [82]	Positive endorsement system that identifies a healthier choice by using a green logo based on the nutritional profile of a product.	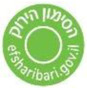
Malaysia [86,93]	An energy-only label based on the Guideline Daily Amount (GDA) (not pictured) and a positive endorsement system that identifies a healthier choice by using a red checkmark logo based on the nutritional profile of a product.	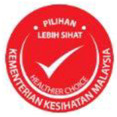
Nigeria [94]	Positive endorsement system that identifies a healthier, “heart-friendly” choice by using a red heart and checkmark logo based on the nutritional profile of a product.	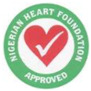
Philippines [86,89]	An energy-only label based on the Guideline Daily Amount (GDA) and a positive endorsement system that identifies a healthier choice by using a green flower logo based on the nutritional profile of a product.	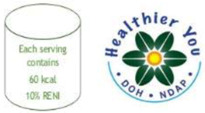
Saudi Arabia [95]	Traffic light label that uses red, amber, and green colors to indicate high, medium, and low levels of fat, saturated fat, total sugars, and salt.	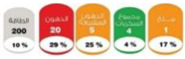
Singapore [96]	Positive endorsement system that identifies a healthier choice by using a red pyramid logo and taglines based on the nutritional profile of a product.	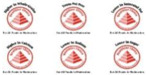
Slovenia [88]	A positive endorsement system that identifies a healthier choice by using a heart symbol logo based on the nutritional profile of a product.	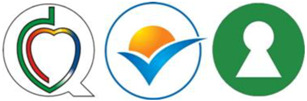
South Africa [97]	Positive endorsement system that identifies a healthier choice by using a red heart logo based on the nutritional profile of a product.	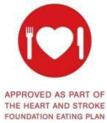
South Korea [98]	Multiple traffic light label options that use red, amber, and green colors to indicate high, medium, and low levels of total fat, saturated fat, total sugars, and sodium. Only recommended for certain children’s foods.	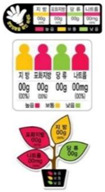
Thailand [99]	Positive endorsement system that identifies a healthier choice by using a colored logo based on the nutritional profile of a product.	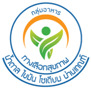
United Arab Emirates [100]	Traffic light label that uses red, amber, and green colors to indicate high, medium, and low levels of fat, saturated fat, sugars, and salt.	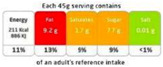
United Kingdom [101]	Traffic light label that uses red, amber, and green colors to indicate high, medium, and low levels of fat, saturated fat, sugars, and salt.	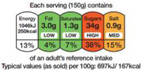
Zambia [102]	Positive endorsement system that identifies a healthier choice by using a colored logo based on the nutritional profile of a product.	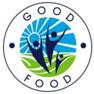
Zimbabwe [89]	Positive endorsement system that identifies a healthier choice by using a red heart logo based on the nutritional profile of a product.	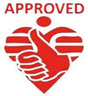

**Table 5:** Binding sites of common natural and artificial sweeteners on the sweet taste receptor.

Sweetener [E-Number]	Compound Classification and Source	Sweet Taste Receptor Binding Site(s)	Bitter Taste Receptor Binding Site(s)
Sucrose [108,109,110,111]	Carbohydrate	Venus flytrap domain of T1R2 and T1R3	N/A
Cyclamates [E952] [108,112,113]	Sulfamic acid derivative,	Transmembrane domain of T1R3	T2R1 and T2R38
Sucralose [E955] [109,110,112,114]	Trichlorinated disaccharide, sugars	Venus flytrap domain of T1R2 and T1R3	Binds to but does not activate: T2R1, T2R4, T2R5, T2R7, T2R8, T2R10, T2R39, T2R41, T2R46
Aspartame [E951] [109,112,115]	Dipeptide, amino acids	Venus flytrap domain of T1R2	Not yet known
Acesulfame-K [E950] [116,117,118]	Sulfamate ester,	Venus flytrap domain of T1R2	T2R43 and T2R44
Saccharin [E954] [118]	Benzoic acid sulfimide		
Stevia and its glycosides (ex. Reb A, Reb M, etc.) [E960] [110,111,112,119,120]	Glycosylated diterpenoid, *Stevia rebaudiana* Bertoni	Venus flytrap domain of T1R2 and T1R3	T2R4 and T2R14
Monk fruit (ex. Mogroside V) [119,121]	Glycosylated triterpenoid, *Siraitia grosvenorii* (Luo Han Guo)	Venus flytrap domain of T1R2 and T1R3	Not yet known
Neohesperidin dihydrochalcone (NHDC) [E959] [108,110,122,123]	Glycoside, citrus fruit	Transmembrane domain of T1R3	Not yet known
Thaumatin [E957] [110,119,124]	Sweet protein, *Thaumatococcus daniellii* (Katemfe)	Cysteine-rich domain of T1R3	Not yet known
Brazzein [124,125]	Sweet protein, Oubli (*Pentadiplandra brazzeana*)	Cysteine-rich domain of T1R3	Not yet known
Monellin [122]	Sweet protein, *Dioscoreophyllum cumminsii*	Venus flytrap domain of T1R2	Not yet known

**Table 7:** Substitution of LNCSs for added sugars is physiologically beneficial.

	Cardiometabolic Outcomes	Gut Microbiome	Food Intake and Satiety Management
**Level of evidence**	Systematic reviews and meta-analyses	Preliminary randomized controlled trials	Emerging in vitro evidence and randomized controlled trial
**Natural LNCSs (such as steviol glycosides, rare sugars)**	positive impact	no effect	decrease
**Artificial LNCSs (such as ace-K, aspartame, sucralose, saccharin)**	positive impact	mixed effects	decrease

LNCSs: low and no-calorie sweeteners.

## Data Availability

Data will be provided upon request. The data presented in this study are available on request from the corresponding author due to legal reason.
